# Osteochondrosis and other lesions in all intervertebral, articular process and rib joints from occiput to sacrum in pigs with poor back conformation, and relationship to juvenile kyphosis

**DOI:** 10.1186/s12917-021-03091-6

**Published:** 2022-01-18

**Authors:** Kristin Olstad, Torunn Aasmundstad, Jørgen Kongsro, Eli Grindflek

**Affiliations:** 1 Faculty of Veterinary Medicine, Department of Companion Animal Clinical Sciences, Equine Section, Norwegian University of Life Sciences, P. O. Box 5003, NO-1432 Ås, Norway; 2 Norsvin SA, Storhamargata 44, 2317 Hamar, Norway

**Keywords:** Helical computed tomography, Juvenile (Scheuermann’s) kyphosis, Neuro-central synchondrosis, Osteochondrosis, Swine, Vascular failure, Wedge vertebra, Zygapophyseal joint

## Abstract

**Background:**

Computed tomography (CT) is used to evaluate body composition and limb osteochondrosis in selection of breeding boars. Pigs also develop heritably predisposed abnormal curvature of the spine including juvenile kyphosis. It has been suggested that osteochondrosis-like changes cause vertebral wedging and kyphosis, both of which are identifiable by CT. The aim of the current study was to examine the spine from occiput to sacrum to map changes and evaluate relationships, especially whether osteochondrosis caused juvenile kyphosis, in which case CT could be used in selection against it. Whole-body CT scans were collected retrospectively from 37 Landrace or Duroc boars with poor back conformation scores. Spine curvature and vertebral shape were evaluated, and all inter-vertebral, articular process and rib joints from the occiput to the sacrum were assessed for osteochondrosis and other lesions.

**Results:**

Twenty-seven of the 37 (73%) pigs had normal spine curvature, whereas 10/37 (27%) pigs had abnormal curvature and all of them had wedge vertebrae. The 37 pigs had 875 focal lesions in articular process and rib joints, 98.5% of which represented stages of osteochondrosis. Five of the 37 pigs had focal lesions in other parts of vertebrae, mainly consisting of vertebral body osteochondrosis. The 10 pigs with abnormal curvature had 21 wedge vertebrae, comprising 10 vertebrae without focal lesions, six ventral wedge vertebrae with ventral osteochondrosis lesions and five dorsal wedge vertebrae with lesions in the neuro-central synchondrosis, articular process or rib joints.

**Conclusions:**

Computed tomography was suited for identification of wedge vertebrae, and kyphosis was due to ventral wedge vertebrae compatible with heritably predisposed vertebral body osteochondrosis. Articular process and rib joint osteochondrosis may represent incidental findings in wedge vertebrae. The role of the neuro-central synchondrosis in the pathogenesis of vertebral wedging warrants further investigation.

**Supplementary Information:**

The online version contains supplementary material available at 10.1186/s12917-021-03091-6.

## Background

In the literature, it is documented that young pigs can develop abnormal curvature of the back during growth [1], but the terminology to describe it is often used imprecisely. In the current report, the term “humpback” will be used to denote abnormal dorsal deviation and the term “dipped back” will be used to denote abnormal ventral deviation of the conformation of the back. The terms “kyphosis, “lordosis” and “scoliosis” will be used when referring to abnormal dorsal, ventral or lateral curvature of the spine, respectively. Dipped and humpback can occur without abnormal spine curvature, in which case the conformations are most often considered postural responses to limb pain [2, 3]. The reported prevalence of humpback is 2.5–11.4% [4] but there are outbreaks where 30–35% of pigs have to be destroyed due to humpback [5, 6]. In one case series, 58% of pigs with humpback showed no clinical signs [2], but humpback is also associated with signs of spinal cord compression ranging from difficulty rising to life-threatening paraplegia [2, 7]. Some authors note other diseases in the herd around the time that humpback manifests, including pneumonia [2, 4]. Slower growth rate is documented in pigs with kyphosis, to the extent that pigs might fail to reach slaughter weight [4]. Holl et al. [8] describes kyphosis without humpback at necropsy and considers that the structural abnormalities could cause difficulty boning out and reduced carcass value. Thus, irrespective of the degree to which humpback causes clinical signs, it is an indication of a problem in the herd and, for sustainable production, the condition must be addressed [4]. Holl et al. [8] estimates the heritability of kyphosis at necropsy at 0.32, meaning that if humpback is appropriately categorised, it should be possible to reduce prevalence through selective breeding.

**Fig. 1:**
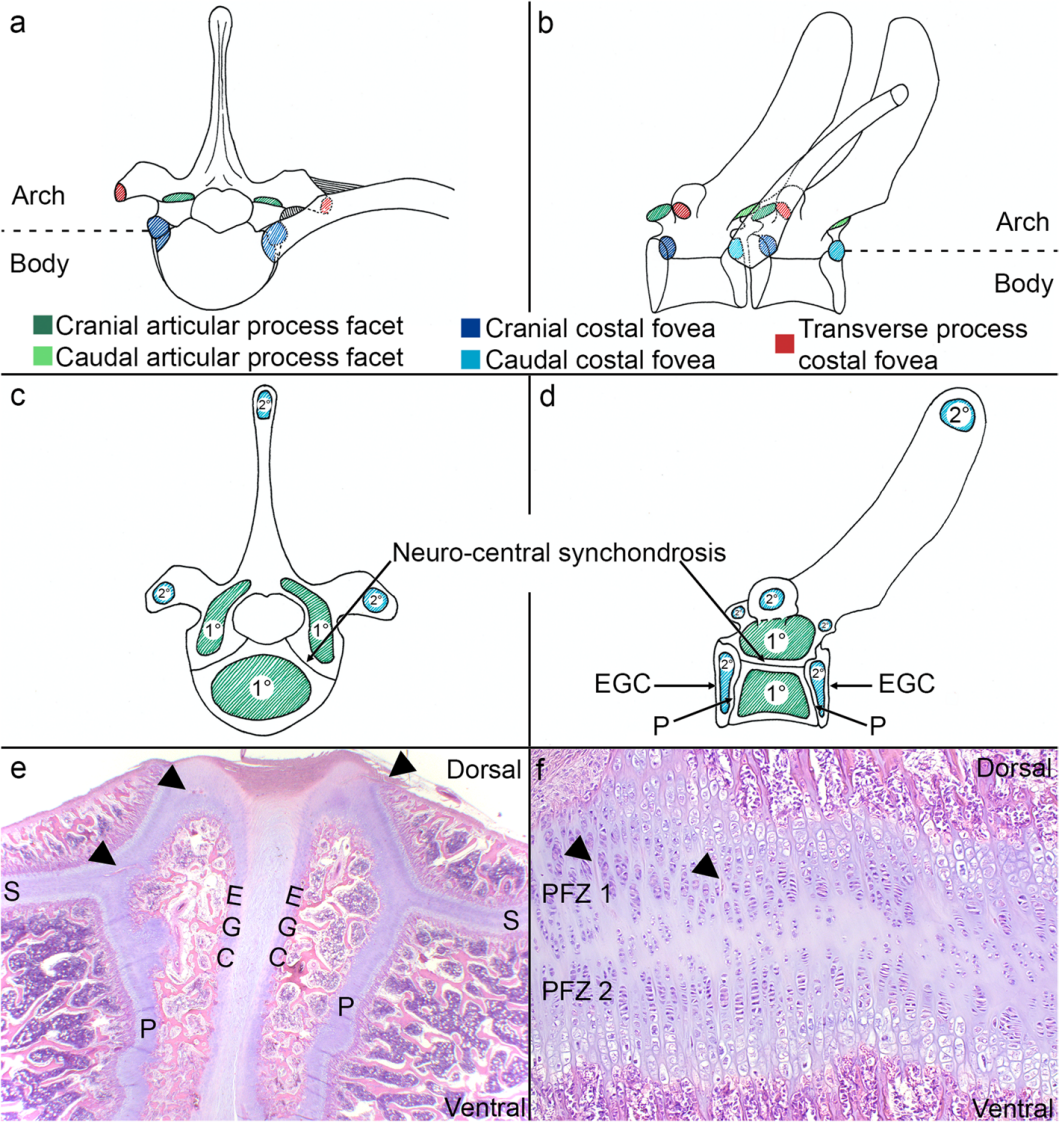
Vertebral anatomy, ossification centres and growth cartilage. **a-b.** Vertebrae consist of a body and a neural arch. **b.** The cranial and caudal articular facets (dark and light green) meet at the articular process joints. **b.** Adjacent vertebral bodies articulate with the head of a rib at the caudal and cranial costal foveae (dark and light blue) to form the costo-vertebral joint. **a-b.** The rib tubercle articulates with the costal fovea of the transverse process (red) to form the costo-transverse joint. **c-d.** The primary ossification centre (1°) is responsible for forming the ventral 2/3 of the vertebral midbody, whereas **d.** the secondary ossification centres (2°) cover the entire cranial and caudal ends of the vertebral body. **c-d.** The body and arch meet at the neuro-central synchondrosis (labelled). **c.** The neural arch forms from a pair of primary ossification centres (1°) located within the left and right demi-arches, respectively, and **c-d.** the spinous process, transverse process and cranial and caudal articular processes have secondary ossification centres (2°). **d-e.** Between primary and secondary ossification centres, there are metaphyseal growth plates or physes (P) and superficial to secondary ossification centres, there is epiphyseal growth cartilage (EGC). **e-f.** The growth cartilage of the physes and synchondrosis (S) contains patent vessels or eosinophilic streaks (arrowheads) representing the cartilage canal blood supply. **f.** The synchondrosis is bidirectional and contains one proliferative zone (PFZ1) contributing to the neural arch and a second proliferative zone (PFZ2) forming the dorsal 1/3 of the vertebral midbody. **a-b.** Tracing of T7 from photograph; **a.** cranial view, **b.** lateral view. **c-d.** Simplified indication of ossification centres from CT scans on top of tracings; **a.** mid-body transverse plane, **b.** lateral view. **e-f.** Para-sagittal histological section from T4-T5 of a 12 kg mixed-breed piglet; **e.** 10x; **f.** 100x magnification, haematoxylin and eosin

With respect to appropriate categorisation, the literature contains anatomical descriptions relevant to pigs of the current examined 146–175-day age range that bears repetition because it is not well-known and repeating it will facilitate reading this report. Vertebrae consist of a body and a neural arch (Fig. 1a-b).

The vertebral body resembles a limb long bone in that there is a primary, diaphyseal ossification centre in the middle (Fig. 1c-d) and one secondary, epiphyseal ossification centre at each of the cranial and caudal ends (Fig. 1d) [1, 9, 10]. Between the primary and secondary ossification centres, there is a metaphyseal growth plate or physis, and between the secondary ossification centre and the intervertebral, fibrocartilaginous disc joint, there is epiphyseal growth cartilage (Fig. 1d) [1, 9, 10]. The secondary ossification centres cover the entire cranial and caudal ends of the vertebral body, but the primary ossification centre is only responsible for forming the ventral 2/3 of the vertebral mid-body (Fig. 1d) [9, 10]. In the region where the neural arch meets the vertebral body, or centrum, there is a structure known as the neuro-central synchondrosis (Fig. 1c-d) [9, 10]. The synchondrosis consists of bidirectional growth cartilage (Fig. 1e-f), thus contributing both to the dorsal 1/3 of the vertebral body and to the neural arch [9, 10]. The neural arch forms from a separate pair of primary ossification centres located within the left and right demi-arches, respectively (Fig. 1c) [9, 10]. The neural arch has several protrusions, most notably the cranial and caudal articular processes that meet at the synovial articular process joints (Fig. 1b) (synonyms: zygapophyseal or facet joints) [9, 10]. The articular processes have secondary ossification centres (Fig. 1d), meaning that like vertebral bodies, they also have physes and sub-articular epiphyseal growth cartilage [9, 10]. In the thoracic segment, adjacent vertebral bodies articulate with the head of a rib at the caudal and cranial costal foveae near the neuro-central synchondrosis to form one costo-vertebral joint (Fig. 1b) [9, 10]. The rib tubercle articulates with the costal foveal of the transverse process to form one costo-transverse joint (Fig. 1a-b) [9, 10], and the transverse process has a secondary ossification centre that is also surrounded by growth cartilage.

Vertebral growth cartilage has a blood supply (Fig. 1e-f), most systematically described in humans [11, 12] and rabbits [13, 14]. This is relevant because in the limbs of pigs, the temporary, end-arterial blood supply to growth cartilage can fail and initiate osteochondrosis [15]. Failure is usually presumed to be heritably predisposed, but bacteraemia from acquired infections can also occlude vessels and trigger the same pathogenesis as heritable, aseptic vascular failure [16, 17]. In sub-articular epiphyseal growth cartilage, vascular failure leads to ischaemic chondronecrosis [15], whereas in the physis, it leads to retention of viable hypertrophic chondrocytes [18] and in both locations, the areas of de-vascularised cartilage resist replacement by bone and cause the focal delay in endochondral ossification that is the definition of osteochondrosis [3, 19]. Articular osteochondrosis can resolve, or progress to osteochondrosis dissecans (OCD) or subchondral bone cysts [20]. Physeal osteochondrosis also resolves [21] but has been associated with valgus/varus angular limb deformities that manifest some months later [19]. There are similarities between the bone angulation associated with physeal osteochondrosis [19] and the vertebral wedging that occurs in juvenile kyphosis, lordosis and scoliosis. Nielsen et al. [1] and Corradi et al. [5] noted that the ventral half of secondary, epiphyseal ossification centres was absent in ventrally wedged vertebrae (excessively short ventral contour) from pigs with juvenile kyphosis, which instead contained dysplastic or degenerated cartilage with failed or obstructed vessels, potentially analogous to the vascular failure that occurs in limb osteochondrosis [15, 18]. Osteochondrosis has been described in the dorsally located articular process joints of pigs [3, 19], but it is not known whether this can cause dorsal wedging (excessively short dorsal contour) and lordosis. As will be discussed, scoliosis has been experimentally induced in pigs [22], but this is usually done through physical tethering and does not give any information about whether spontaneous lateral wedging (excessively short left or right contour) is likely to occur by any heritable, osteochondrosis-like mechanism. Computed tomography (CT) is used to quantify lean tissue for boar selection in some pig populations [23], and it has also been validated as a screening tool for osteochondrosis in limb joints [24] and physes [18]. In the literature, there is an understandable tendency to focus on smaller parts of spine or vertebrae at a time. We had access to whole-body CT scans and in this initial study, we wanted to evaluate as much of the spine as possible to generate a representative overview of lesions and relationships upon which to base future research and selection efforts.

The aim of the current study was to evaluate the spine from occiput to sacrum to map changes and evaluate relationships, especially whether abnormal curvature was due to osteochondrosis, in which case CT could be used in selection against it.

## Results

### Number of pigs, vertebrae, transitional vertebrae and joints assessed

There were 37 pigs and the conformation score, weight and breed of each pig are listed in Table 1. Pigs that received medical treatments were labelled with an asterisk, and all available treatment details are listed in Supplemental Table 1.

**Table 1:** Pigs

Pig	Conformation	Score	Weight	Breed	Cervical vertebrae	Thoracic vertebrae	Lumbar vertebrae	Sacral vertebrae	Sum	Anticlinal vertebra	Rib joints merge at	Transitional vertebrae	Rib
1	Dipped back	Severe	100 kg	Landrace	7	15	6	4	32	10	11	–	–
2^*^	Dipped back	Severe	100 kg	Landrace	7	16	6	4	33	13	11	T16	Both sides
3	Dipped back	Severe	100 kg	Landrace	7	17	6	4	34	14	11	–	–
4	Dipped back	Severe	100 kg	Landrace	7	16	5	4	32	12	12	T16	Left only
5	Dipped back	Severe	100 kg	Landrace	7	16	6	4	33	11	11	–	–
6^*^	Dipped back	Severe	100 kg	Landrace	7	16	6	4	33	12	12	T16	Both sides
7^*^	Dipped back	Severe	100 kg	Landrace	7	17	6	4	34	13	11	–	–
8	Dipped back	Severe	100 kg	Duroc	7	15^a^	6	4	31	11	12	–	–
9	Dipped back	Severe	100 kg	Landrace	7	15	6	4	32	13	13	–	–
10^*^	Dipped back	Severe	100 kg	Landrace	7	16	6	4	33	13	15	–	–
11	Dipped back	Severe	100 kg	Landrace	7	16	6	4	33	14	11	–	–
12^*^	Dipped back	Severe	100 kg	Landrace	7	15	6	4	32	12	11	–	–
13	Dipped back	Severe	120 kg	Landrace	7	16	6	4	33	12	12	–	–
14	Dipped back	Severe	120 kg	Duroc	7	15	5	4	31	12	11	T15	Both sides
15^*^	Dipped back	Severe	120 kg	Landrace	7	16	6	4	33	13	12	–	–
16^*^	Dipped back	Severe	120 kg	Landrace	7	17	6	4	34	13	11	T17	Left only
17	Dipped back	Severe	120 kg	Duroc	7	15	6	4	32	11	11	–	–
18^*^	Humpback Dipped back	Severe Severe	120 kg	Landrace	7	16	6	4	33	12	11	–	–
19	Humpback	Moderate	120 kg	Landrace	7	16	6	4	33	12	12	–	–
20	Humpback	Moderate	120 kg	Landrace	7	15	6	4	32	11	11	–	–
21	Humpback	Moderate	120 kg	Duroc	7	15	6	4	32	13^b^	12	–	–
22^*^	Humpback	Moderate	120 kg	Landrace	7	17	5	4	33	13	12	T17	Both sides, but left > right
23^*^	Humpback	Moderate	120 kg	Duroc	7	16	6	4	33	11	11	–	–
24	Humpback	Moderate	120 kg	Duroc	7	16	6	4	33	11	11	–	–
25^*^	Humpback	Moderate	120 kg	Duroc	7	16	6	4	33	12	12	–	–
26^*^	Humpback	Moderate	120 kg	Landrace	7	15	6	4	32	13	11	–	–
27^*^	Humpback	Moderate	120 kg	Landrace	7	16	6	4	33	13	12	–	–
28^*^	Humpback	Moderate	120 kg	Duroc	7	15	6	4	32	11	11	–	–
29^*^	Humpback	Moderate	120 kg	Landrace	7	16	6	4	33	13	12	T16	Both sides, but right > left
30^*^	Humpback	Moderate	120 kg	Landrace	7	16	6	4	33	12	12	–	–
31	Humpback	Moderate	120 kg	Duroc	7	15	6	4	32	12	11	–	–
32	Humpback	Moderate	120 kg	Duroc	7	15	6	4	32	12	11	–	–
33^a^	Humpback	Moderate	120 kg	Duroc	7	14	6	4	31	12	12	–	–
34	Humpback	Moderate	120 kg	Landrace	7	16	6	4	33	13	11	–	–
35	Humpback	Moderate	120 kg	Landrace	7	16	5	4	32	12	12	T16	Both sides, but right > left
36	Humpback	Moderate	120 kg	Duroc	7	15	6	4	32	12	12	–	–
37^*^	Humpback	Moderate	120 kg	Duroc	7	14	6	4	31	12	Never	–	–
Sum	17 dipped back 19 humpback 1 both	19 severe 19 moderate	37 pigs: 12 pigs 100 kg 25 pigs 120 kg	24 Landrace 13 Duroc	259	579	218	148	1204	451	417	8 pigs	14 ribs
Mean					7	15.6	5.9	4	32.5	12.2	11.6		
Median					7	16	6	4	33	12	11		
Min.					7	14	5	4	31	10	11		
Max.					7	17	6	4	34	14	15		

Number of joints evaluated: 740 cervical joints (37 atlanto-occipital + 259 intervertebral + 444 articular process joints) + 2407 thoracic joints (579 intervertebral + 1158 articular process + 670 [302 separate + 368 merged] rib joints) + 654 lumbar joints (218 intervertebral + 436 articular process joints) = 3801 joints

^*^Pigs that received medical treatments are labelled with an asterisk; all available details are summarised in Supplemental Table 1

^a^In pig 8, T13 and T14 were fused to a block vertebra but were counted as two vertebrae because there were two primary ossification centres in the body of the block vertebra

^b^Vertebral wedging may have interfered with correct identification of the anticlinal vertebra in pig 21

**Fig. 2:**
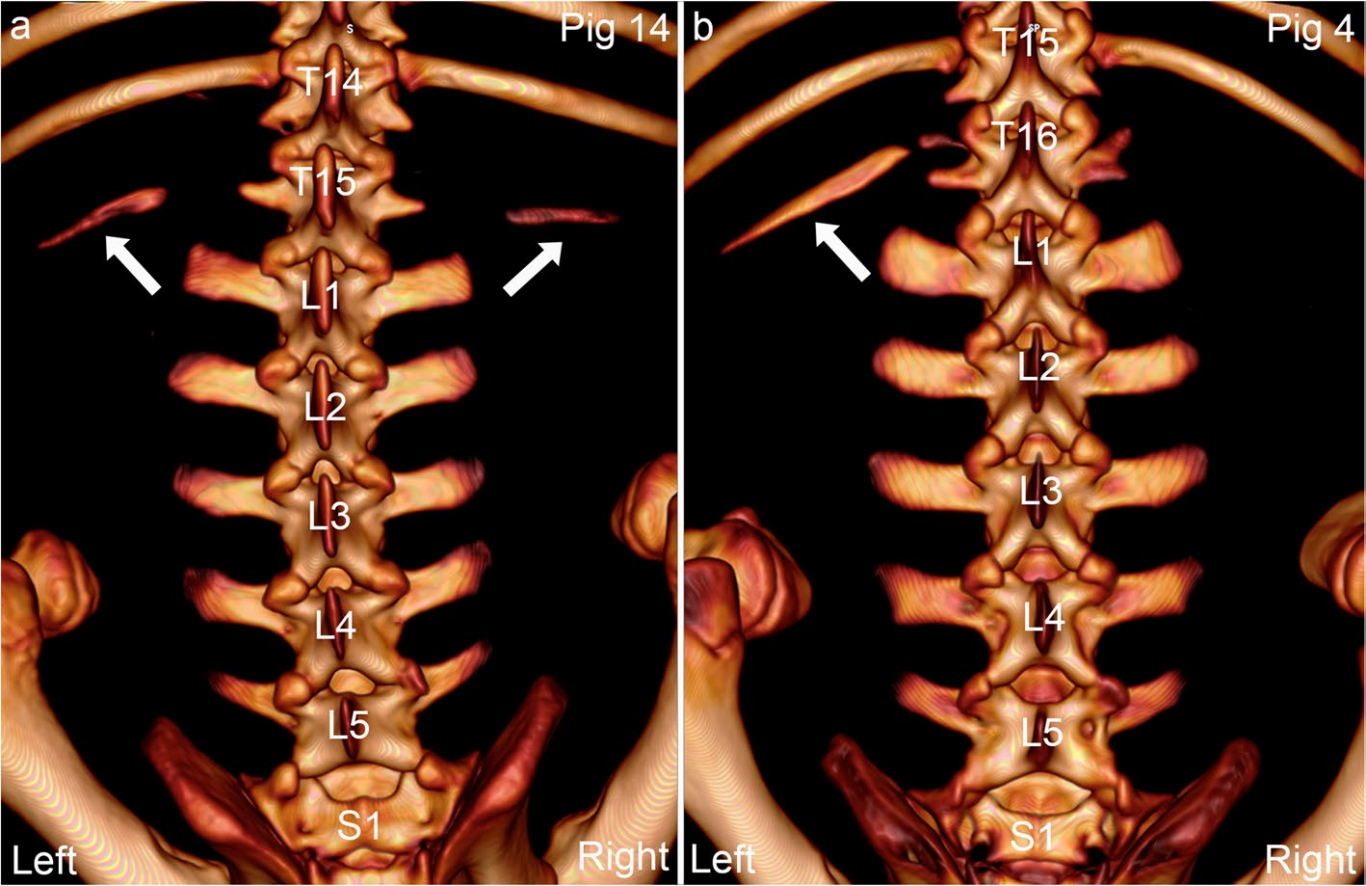
Transitional vertebrae with characteristics of both the cranially and caudally adjacent segments. **a.** Pig 14 has a transitional vertebra T15 at the thoraco-lumbar junction with both a left and a right rib (arrows), and five lumbar vertebrae. The spine curvature is normal.**b.** Pig 4 has a transitional vertebra T16 at the thoraco-lumbar junction that is asymmetric in the sense that it only has one rib on the left side (arrow), but spine curvature is normal. **a-b.** 3D model, dorsal view

A total of 1204 vertebrae were counted in the 37 pigs, corresponding to a median of 33 vertebrae per pig and a formula of C7 + T16 + L6 + S4 (Table 1). The number of thoracic and sacral vertebrae was fixed. The number of thoracic vertebrae ranged from 14 to 17 (mean: 15.6). The number of lumbar vertebrae ranged from 5 to 6, and all four pigs with five lumbar vertebrae had transitional vertebrae (Table 1). Transitional vertebrae only occurred at the thoraco-lumbar junction, represented thoracisation of lumbar vertebrae/lumbar ribs (Fig. 2) and were counted with thoracic vertebrae. Transitional vertebrae occurred in 8/37 (22%) pigs: the four pigs with five lumbar vertebrae, and a further four pigs with six lumbar vertebrae (Table 1). Spine curvature was normal at transitional vertebrae, even in pigs 4 (Fig. 2b) and 16 where the transitional vertebra was asymmetric in terms of having only one rib. Rib head and tubercle joints tended to merge into a single articulation from T11, thus a total of 3801 joints were assessed in the 37 pigs (mean: 103 joints per pig; Table 1 legend).

### Pigs with abnormal spine curvature and abnormal vertebrae

Twenty-seven of the 37 (73%) pigs had normal spine curvature and 10/37 (27%) pigs had abnormal curvatures in the thoracic segment, listed in Table 2. The 10 pigs had a total of 22 abnormal curvatures. Kyphosis was the most common abnormal curvature, present in eight pigs. Pig 8 had just kyphosis and pig 34 had just lordosis, whereas the remaining eight pigs had multiple abnormalities, of which kyphosis and lordosis were the most common combination, present in four pigs (Table 2). Pig 7* was the only pig that had all three abnormalities kyphosis, lordosis and scoliosis.

**Table 2:** Ten pigs with abnormal curvature

Pig	Abnormal curvature	Wedge vertebra	Vertebral lesions	Articular process and rib joint lesions	Lesion and curvature match
1	Kyphosis	T4 ventral wedge	No	No	No
	Lordosis	T10 dorsal wedge	No	Left and right articular process OC^a^	Dorsal lesions and wedge match
2^*^	Kyphosis	T5 ventral wedge	No	No	No
	Lordosis	T7 dorsal wedge	No	No	No
7^*^	Kyphosis	T6 ventral wedge	No	No	No
	Lordosis	T7 dorsal wedge and fracture	Neuro-central synchondrosis fracture	No	Dorsal lesions and wedge match
	Lordosis, scoliosis	T8 dorsal and left wedge	No	No	No
8	Kyphosis	T14–15 ventral wedge block vertebra and right shift	T14 left cranio-ventral cyst; T15 left cranio-ventral OC; T15 left caudo-ventral sclerosis; multiple transverse process lesions	Multiple articular process joint lesions	Ventral lesions and wedging match, but also dorsal lesions
11	Kyphosis, scoliosis	T16 ventral and right wedge	Right caudo-ventral right OC	No	Right ventral lesions and wedge match
12^*^	Kyphosis	T3 ventral wedge	No	No	No
	Lordosis	T8 dorsal wedge	No	No	No
15^*^	Kyphosis	T4–5 mild ventral wedge	No	No	No
	Lordosis	T8–9 mild dorsal wedge	No	T8 left articular process OC; T9 left articular process OCD^b^	Dorsal lesions and wedge match; left side mismatch
18^*^	Lordosis, scoliosis	T9 dorsal and left wedge	No	No	No
21	Kyphosis	T13 ventral wedge	Left cranio-ventral OCD	No	Ventral lesion and wedge match; left side mismatch
	Kyphosis, scoliosis	T14 ventral and left wedge, spondylosis	Midline cranio-ventral OCD and spondylosis; left caudo-ventral OC	No	Ventral and left lesions and wedge match
	Kyphosis	T15 ventral wedge	Mid-to-left caudo-ventral cyst	No	Ventral lesion and wedge match; left side mismatch
34	Lordosis	T9 dorsal wedge	No	Right side articular process joint double line	Dorsal lesion and wedge match; right side mismatch
10 pigs	22 curvatures: 10 kyphosis 8 lordosis 4 scoliosis	21 wedge vertebrae: 12 ventral wedge 9 dorsal wedge 4 lateral wedge	10 pigs: 6 pigs no lesions 4 pigs lesions	10 pigs: 7 pigs no lesions 3 pigs lesions	10 pigs: 3 pigs no match 4 pigs match 3 pigs partial match

^*^Pigs that received medical treatments are labelled with an asterisk

^a^*OC* Osteochondrosis

^b^*OCD* Osteochondrosis dissecans

At the site of abnormal curvature, the 10 pigs had 21 wedge vertebrae (Table 2). Ventral wedge vertebrae T13 and T14 in pig 8 represented a block vertebra but were counted as two vertebrae because there were two primary ossification centres within the body of the block vertebra (see below). Left or right wedging was only observed in combination with dorsal or ventral wedging. Ventral wedging was most common, occurring in 12 vertebrae, whereas dorsal wedging occurred in nine vertebrae and the four times that lateral wedging occurred were distributed equally as two times in ventral wedge and two times in dorsal wedge vertebrae (Table 2). Three pigs had one wedge vertebra, whereas seven pigs had from 2 to 4 wedge vertebrae each. Pig 15* was the only pig that had four wedge vertebrae.

### Focal lesions in articular process and rib head and tubercle joints – per pig

The 37 pigs had 875 focal lesions in their articular process and rib head and tubercle joints, and the distribution of lesions per pig, important for selection, is shown in Table 3. Please note that the sum of one-sided and multi-sided lesions meant that the total number of affected articulations was 662 (Table 3). The pigs had a mean of 23.6 lesions each (range: 8–94), affecting a mean of 12.5 vertebrae per pig (range: 6–24). When correcting for variation in vertebral number, this corresponded to a mean percentage of 44.1% affected vertebrae per pig (range: 20–88.9%; Table 3).

**Table 3:** Distribution of the 875 articular process and rib joint lesions among the 37 pigs

Pig	Whole spine			Cervical segment							
	Lesions	Total: affected vertebrae	Percentage	Total: affected vertebrae	Lesions	Symmetric: asymmetric lesions	One-sided: two-sided lesions	OC^a^	OCD^b^	Cyst	Other
1	24	28:13	46.4%	7:2	3	0:3	1:2	1	2	–	–
2^*^	11	28:7	25%	7:0	–	–	–	–	–	–	–
3	11	30:9	30%	7:3	4	0:4	0:4	4	–	–	–
4	20	28:16	57.1%	7:2	2	0:2	2:0	1	–	1	–
5	15	29:9	31%	7:0	–	–	–	–	–	–	–
6^*^	29	29:15	51.7%	7:2	3	0:3	1:2	3	–	–	–
7^*^	8	30:6	20%	7:0	–	–	–	–	–	–	–
8	14	28:11	39.3%	7:0	–	–	–	–	–	–	–
9	16	28:12	42.9%	7:5	6	0:6	2:4	4	2	–	–
10^*^	32	29:15	51.7%	7:0	–	–	–	–	–	–	–
11	10	29:7	24.1%	7:3	4	0:4	2:2	4	–	–	–
12^*^	13	28:8	28.6%	7:1	1	0:1	1:0	1	0	0	0
13	30	29:14	48.3%	7:3	4	0:4	0:4	4	–	–	–
14	19	27:10	37%	7:3	7	4:3	3:4	6	–	1	–
15^*^	36	29:19	65.5%	7:5	8	4:4	2:6	4	4	–	–
16^*^	21	30:14	46.7%	7:2	4	0:4	2:2	2	–	–	2 OA^c^
17	8	28:6	21.4%	7:1	1	0:1	1:0	1	–	–	–
18^*^	11	29:6	20.7%	7:1	1	0:1	1:0	–	1	–	–
19	22	29:13	44.8%	7:3	3	0:3	3:0	3	–	–	–
20	18	28:10	35.7%	7:3	5	0:5	3:2	3	–	–	2 OA
21	20	28:8	28.6%	7:2	2	0:2	2:0	1	1	–	–
22^*^	36	29:17	58.6%	7:2	4	2:2	4:0	3	1	–	–
23^*^	13	29:9	31%	7:0	–	–	–	–	–	–	–
24	12	29:7	24.1%	7:2	2	0:2	2:0	2	–	–	–
25^*^	17	29:12	41.4%	7:3	4	2:2	2:2	4	–	–	–
26^*^	26	28:16	57.1%	7:3	4	0:4	2:2	3	1	–	–
27^*^	23	29:17	58.6%	7:4	4	0:4	2:2	3	1	–	–
28^*^	44	28:19	67.9%	7:2	5	4:1	3:2	3	2	–	–
29^*^	28	29:16	55.2%	7:4	6	2:4	4:2	6	–	–	–
30^*^	22	29:11	37.9%	7:2	3	2:1	1:2	3	–	–	–
31	26	28:13	46.4%	7:2	2	0:2	0:2	2	–	–	–
32	35	28:20	71.4%	7:3	4	0:4	4:0	3	1	–	–
33^*^	16	27:8	29.6%	7:0	–	–	–	–	–	–	–
34	17	29:11	37.9%	7:1	2	2:0	2:0	2	–	–	–
35	30	28:16	57.1%	7:2	2	0:2	0:2	2	–	–	–
36	48	28:20	71.4%	7:5	8	2:6	6:2	6	1	1	–
37^*^	94	27:24	88.9%	7:4	6	4:2	2:4	6	–	–	–
SUM	875	1056: 464	1631%	259:80 30.9%	114	28:86	60:54	90	17	3	4
Mean	23.6	28.5: 12.5	44.1%	7:2.2	3.1	24.6%: 75.4%	52.6%: 47.4%	78.9%	14.9%	2.6%	3.6%
Median	20	29:12	42.9%	7:2	3						
Min	8	27:6	20%	0	0						
Max	94	29:24	88.9%	5	8						
	1.9 lesions per affected vertebra			1.4 lesions per affected vertebra							

Pig	Thoracic segment								Lumbar segment							
	Total: affected vertebrae	Lesions	Symmetric: asymmetric lesions	One-sided: two-sided: three-sided lesions	OC	OCD	Cyst	Other	Total: affected vertebrae	Lesions	Symmetric: asymmetric lesions	One-sided: two-sided lesions	OC	OCD	Cyst	Other
1	15:6	8	2:6	8:0:0	6	2	–	–	6:5	13	12:1	5:8	13	–	–	–
2^*^	16:4	4	0:4	4:0:0	4	–	–	–	6:3	7	4:3	5:2	4	2	1	–
3	17:2	2	0:2	2:0:0	2	–	–	–	6:4	5	2:3	3:2	5	–	–	–
4	16:9	9	0:9	9:0:0	3	5	1	–	5:5	9	6:3	7:2	7	2	–	–
5	16:7	11	2:9	9:2:0	6	3	–	2 stu-mp	6:2	4	4:0	4:0	3	1	–	–
6^*^	16:7	11	4:7	11:0:0	7	3	1	–	6:6	15	10:5	5:10	15	–	–	–
7^*^	17:1	2	2:0	2:0:0	2	–	–	–	6:5	6	2:4	4:2	5	1	–	–
8	15:8	10	2:8	4:6:0	1	9	–	–	6:3	4	0:4	4:0	3	1	–	–
9	15:6	8	2:6	8:0:0	8	–	–	–	6:1	2	0:2	2:0	1	–	1	–
10^*^	16:9	14	2:12	10:4:0	12	1	1	–	6:6	18	12:6	6:12	12	6	–	–
11	16:2	2	0:2	2:0:0	2	–	–	–	6:2	4	0:4	2:2	3	–	1	–
12^*^	15:3	4	0:4	4:0:0	1	2	1	–	6:4	8	8:0	8:0	6	2	–	–
13	16:5	7	0:7	5:2:0	5	2	–	–	6:6	19	16:3	7:12	14	5	–	–
14	15:2	2	0:2	2:0:0	–	–	2	–	5:5	10	8:2	10:0	9	1	–	–
15^*^	16:8	9	0:9	9:0:0	5	3	1	–	6:6	19	14:5	5:14	14	1	4	–
16^*^	17:6	7	0:7	5:2:0	5	2	–	–	6:6	10	6:4	10:0	8	2	–	–
17	15:3	4	0:4	2:2:0	1	2	1	–	6:2	3	0:3	3:0	1	2	–	–
18^*^	16:3	3	0:3	3:0:0	2	–	1	–	6:2	7	4:3	5:2	3	3	1	–
19	16:4	5	2:3	5:0:0	3	2	–	–	6:6	14	10:4	8:6	13	1	–	–
20	15:2	2	0:2	2:0:0	–	2	–	–	6:5	11	6:5	5:6	8	3	–	–
21	15:0	–	–	–	–	–	–	–	6:6	18	14:4	6:12	6	11	1	–
22^*^	17:10	16	8:8	12:4:0	6	7	3	–	5:5	16	12:4	2:14	11	3	2	–
23^*^	16:7	9	4:5	9:0:0	3	3	3	–	6:2	4	2:2	2:2	2	1	1	–
24	16:1	1	0:1	1:0:0	–	1	–	–	6:4	9	6:3	5:4	4	2	1	2 OA
25^*^	16:4	4	0:4	4:0:0	2	1	1	–	6:5	9	4:5	3:6	7	2	–	–
26^*^	15:7	8	0:8	6:2:0	4	3	1	–	6:6	14	4:10	2:12	5	9	–	–
27^*^	16:7	9	0:9	7:2:0	5	3	1	–	6:6	10	6:4	8:2	10	–	–	–
28^*^	15:11	20	8:12	8:12:0	10	9	1	–	6:6	19	18:1	5:14	7	8	4	–
29^*^	16:7	10	0:10	5:2:3	9	1	–	–	6:5	12	8:4	4:8	7	5	–	–
30^*^	16:4	5	2:3	5:0:0	4	–	1	–	6:5	14	10:4	10:4	3	6	5	–
31	15:5	6	0:6	6:0:0	4	1	1	–	6:6	18	12:6	6:12	7	11	–	–
32	15:12	18	4:14	5:10:3	12	4	2	–	6:5	13	10:3	3:10	5	6	2	–
33^*^	14:4	4	0:4	2:2:0	1	2	1	–	6:4	12	10:2	6:6	10	2	–	–
34	16:4	4	0:4	2:2:0	4	–	–	–	6:6	11	4:7	3:8	9	–	2	–
35	16:11	23	8:15	10:10:3	15	1	2	5 Misc^d^	5:3	5	2:3	3:2	5	–	–	–
36	15:10	27	8:19	4:8:15	20	7	–	–	6:5	13	6:7	5:8	7	5	1	–
37^*^	14:14	73	42:31	17:56:0	62	10	1	0	6:6	15	10:5	7:8	11	3	1	–
SUM	579:215 37.1%	361	102:259	209:128:24	236	91	27	7	218:169 77.5%	400	262:138	188: 212	263	107	28	2
Mean	15.6:5.8	9.8	28.3%:71.7%	57.9%: 35.5%: 6.6%	65.4%	25.2%	7.5%	1.9%	5.9:4.6	10.8	65.5%: 34.5%	47%: 53%	65.8%	26.8%	7%	0.4%
Median	16:6	7							6:5	11						
Min	14:0	0							5:1	2						
Max	17:14	73							6:6	19						
	1.7 lesions per affected vertebra								2.4 lesions per affected vertebra							

One-sided: 457 lesions/articulations. Two-sided: 394 lesions/197 articulations. Three-sided: 24 lesions/8 articulations. Total: 875 lesions/662 articulations

^*^Pigs that received medical treatments are labelled with an asterisk

^a^*OC* Osteochondrosis

^b^*OCD* Osteochondrosis dissecans

^c^*OA* Osteoarthritis

^d^*Misc* Miscellaneous

Seven pigs were negative in the cervical segment and one pig was negative in the thoracic segment, but all pigs had two or more lesions in at least one vertebra of the lumbar segment (Table 3). In absolute numbers, there were 114 cervical lesions, 361 thoracic lesions (189 articular process and 172 rib joint lesions) and 400 lumbar lesions. In relative terms, there were 30.9% affected vertebrae in the cervical segment, 37.1% affected vertebrae in the thoracic segment and 77.5% affected vertebrae in the lumbar segment. This translated to a mean of 1.4 lesions, 1.7 lesions and 2.4 lesions per affected vertebra in the cervical, thoracic and lumbar segments, respectively, thus the lumbar segment was over-represented in absolute number of lesions, percentage of affected vertebrae and number of lesions per affected vertebra (Table 3).

For the entire spine, 39.5% of lesions were left-right symmetric and 60.5% were asymmetric (averaged from Table 3), but there were ~ 72–75% asymmetric lesions in the cervical and thoracic segments, and only 34.5% asymmetric lesions in the lumbar segments, thus asymmetry could be greater in some segments than the overall spine percentage suggested. Conversely, a mean 52.2% of lesions were one-sided (cranial, rib or caudal side only; Table 4), and this percentage stayed within +/− 8% of 50% for all segments (Table 4).

**Table 4:** Distribution of the 875 articular process and rib joint lesions among the 30 articulation levels summary. The raw data for this table are in Supplemental Table 2

Segment	Articulation levels	Lesions	Left: right side of the pig	Mean lesions per articulation level	Minimum	Maximum	Median lesions	Cranial side only	Rib side only	Caudal side only	Multiple sides
Cervical	7	114	58:56 50.9%:49.1%	16.3	7 Atlanto-occipital joint	26 C3–4	17	26 22.8%	–	34 29.8%	54 47.4%
Thoracic	16	361^a^	161:200 44.6%:55.4%	22.6	11 T6–7	37 T3–4	23.5	148 41%	30 8.3%	31 8.6%	152 42.1%
Lumbar	7	400	194:206 48.5%:51.5%	57.1	24 L6-S1	73 L2–3	57	158 39.5%	–	30 7.5%	212 53%
Whole spine	30	875	413:462 47.2%:52.8%	29.2	7	73	25	332 37.9%	30 3.4%	95 10.8%	418 47.8%

^a^189 lesions in articular process joints and 172 lesions in rib head and tubercle joints

**Fig. 3:**
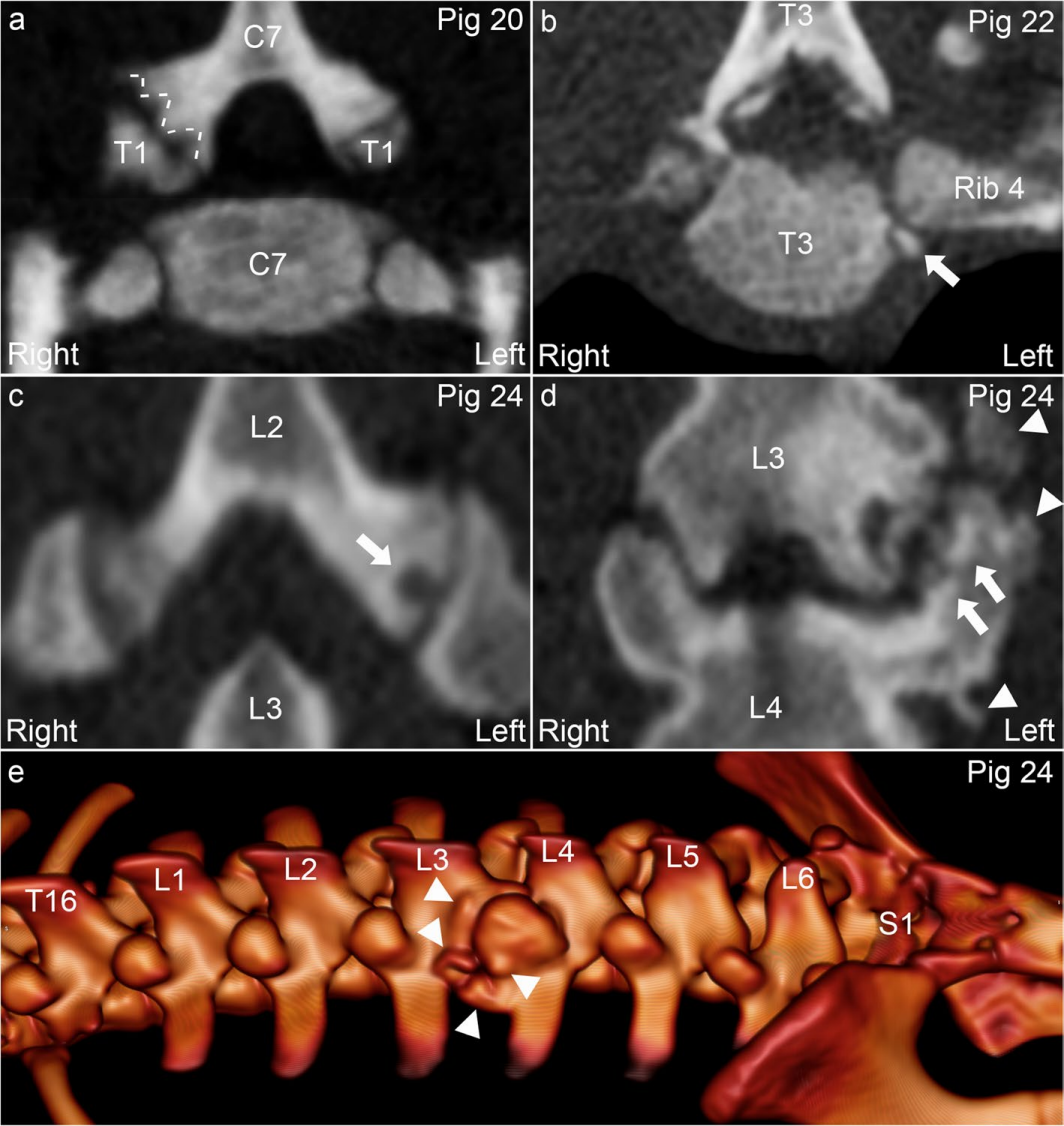
Articular process and rib joint osteochondrosis, osteochondrosis dissecans (OCD), cyst and osteoarthritis. **a.** Pig 20. There is a multi-focal, sharply demarcated, uniformly hypodense defect (dashed lines) categorised as a multi-lobulated osteochondrosis lesion in the right caudal articular process of C7. **b.** Pig 22. There is a mineral hyperdense body (arrow) categorised as an OCD lesion in the joint between the caudal costal facet of T3 and the head of the fourth rib on the left side. **c.** Pig 24. There is a roughly spherical defect (arrow) categorised as a cyst in the left caudal articular process of L2. **d-e.** Pig 24. The joint space is irregular, and there is subchondral bone sclerosis (arrows) and periarticular osteophytes (arrowheads) categorised as osteoarthritis of the left articular process joint of L3-L4. **a-b.** Transverse plane. **c-d.** Dorsal plane. **e.** 3D model, left and slightly left-dorsal-to-right-ventral oblique view

The 875 lesions comprised 589 osteochondrosis lesions (Fig. 3a), 215 OCD lesions (Fig. 3b), 58 cysts (Fig. 3c) (sum: 862/98.5%) and 13 (1.5%) miscellaneous other lesions (Table 3). The 13 other lesions included two cervical and one lumbar articular process joint with osteoarthritis (Fig. 3e-f) (two-sided; six lesions), one articular process joint where both sides were shortened and widened (two “stumps”) potentially representing deformity or callus, and five miscellaneous, atypical lesions in three articular process and rib joints of pig 35: one step deformity (two-sided), one double line and one “flake” of hyperdense material in the joint space (two-sided; Table 3). The proportion of osteochondrosis lesions was higher at 78.9% in the cervical, compared to 65.4 and 65.8% in the thoracic and lumbar segments, respectively.

### Focal lesions in articular process and rib head and tubercle joints – per articulation level

The distribution of the 875 lesions among the 30 articulation levels (articular process and rib joints combined at each vertebral junction), important for predilection and pathogenesis, is summarised in Table 4, and the raw data for Table 4 are in Supplemental Table 2. Not all pigs had T15–17 or L1, but with the reservation that these articulation levels were not representative, the range of lesions per level was from 7 to 73 lesions (mean: 29.2). The least affected level with seven lesions was the atlanto-occipital joint, whereas the most affected level within the cervical segment was C3–4 with 26 lesions. The most affected level in the thoracic segment was T3–4 with 37 lesions, whereas L2–3 was the most affected level both within the lumbar segment and the spine overall with 73 lesions (Table 4).

When shown per location (Table 4) as opposed to per pig (Table 3), it was clear that whether the left or right side of the pig was affected remained within +/− 6% of 50% in all segments, whereas for single-sided lesions, the caudal side was affected 8% more often than the cranial side in the cervical segment and the cranial side was affected ~ 32% more often than the caudal side in the thoracic and lumbar segments, respectively.

### Focal lesions in parts of vertebrae other than articular process and rib joints

Five pigs had focal lesions in parts of 13 vertebrae other than the articular process and rib joints, detailed in Table 5. There were 21 lesions, comprising 11 osteochondrosis lesions and 10 other, variably osteochondrosis-related lesions.

**Table 5:** Detailed list of focal vertebral lesions in five pigs

Pig	Vertebra	Aspect/region	Clockface location^a^	Growth cartilage	Description (number indicates where lesion centred on clockface)	Diagnosis
5	C6	Caudo-dorsal, but cranial to metaphyseal growth plate	11–12 o’clock	Neuro-central synchondrosis	Hypodense line outlining ovoid mineralised body towards floor of vertebral canal	OCD^b^
	C6	Caudal, entire metaphyseal growth plate	Entire clockface	Metaphyseal growth plate	Thin and heterogeneous density	“Physitis”
	C6	Right transverse process	Not applicable	In bone between metaphyseal growth cartilage and synchondrosis	Circular hypodense defect surrounded by bone	Cyst
	C6-C7	Caudo- and cranio-ventral	5–7 o’clock	Epiphyseal growth cartilage	Connected by small bony bridge	Spondylosis
7^*^	C7	Dorsal, entire cranio-caudal length	10–2 o’clock	Neuro-central synchondrosis	Gradual widening to 2–3 times as thick caudally	Type I Salter-Harris fracture
8^c^	C6	Left transverse process	Not applicable	Transverse process unknown growth cartilage	Small and apparently fused with the transverse process of C7. The left transverse process of C5 matched the right transverse process of C6 in size.	Transverse process de−/malformation/ “transposition”
	T13	Left caudo-ventral	4–6	Block vertebra: growth cartilage absent	5–5.30: three mixed-density cones and one larger hemispherical defect 6–7: sclerosis	Osteochondrosis with repair (filling with bone, sclerosis)
	T14	Left cranio-ventral	4–7	Block vertebra: growth cartilage absent	4–6: sclerosis 6–7: spherical defect	Cyst with repair (sclerosis)
	T15	Left cranio-ventral	5.30–6.30	Epiphyseal and metaphyseal growth cartilage	Three small mixed-density cones	Osteochondrosis with repair (filling with bone)
	T15	Left caudo-ventral	5–6.30	Epiphyseal bone	Sclerosis opposite defect in L1	Old lesion scar or response to L1 lesion?
	L1	Left cranio-ventral	4–6	Epiphyseal and metaphyseal growth cartilage	Two conical defects	Osteochondrosis
11	T16	Right caudo-ventral	5–8	Epiphyseal and metaphyseal growth cartilage	5: V-shaped defect 6: two small, hemispherical defects 6–8: two larger hemispherical defects Left lateral abaxial margin: sclerotic margin Further caudo-dorsally: two mixed-density conical defects	Osteochondrosis with secondary repair (filling with bone, sclerotic rim)
21	T13	Left cranio-ventral	4–6	Epiphyseal and metaphyseal growth cartilage	Single-lobe defect with mineralised, OCD-like body, but represents reparative ossification centre from caudal and abaxial to defect	OCD with secondary repair (mineralised body represents repair)
	T14	Midline cranio-ventral	3–9.30	Epiphyseal and metaphyseal growth cartilage	4: stair-step defect 5: small cone with sclerotic rim 6: small cone 8: stair-step defect Mineralised bodies: appear as three separate bodies in individual slices but are confluent in 3D rendering.	Osteochondrosis with repair (sclerotic rim, mineralised body represents repair)
	T14	Left caudo-ventral	3.30–4.30 and 7	Epiphyseal and metaphyseal growth cartilage	4: two mixed-density lobes, sclerotic rim 7: small, square lobe; also smooth, linear mineralisation (in ligament?) extending in arc from cranio-ventral T14 towards caudo-ventral T13.	Osteochondrosis with repair (filling with bone), also spondylosis
	T15	Mid-to-left caudo-ventral	4–8	Epiphyseal and metaphyseal growth cartilage	5.15–30: Two-step stair lesion, spherical with stalk to growth cartilage, with mineralised body advancing from cranio-ventral and around lesion laterally	Cyst with repair (ossification advancing around lesion)

^*^Pigs that received medical treatments are labelled with an asterisk

^a^Clockface location refers to location if superimposing a clockface on transverse plane vertebral body images viewed in the cranio-caudal direction

^b^*OCD* Osteochondrosis dissecans

^c^Pig 8 five transverse (costal) process lesions: T11 left transverse process osteophytes; T12 right transverse process absent; T14 left transverse process thin, irregular shape, projects laterally; T14 right transverse process thick, irregular, projects laterally and returns to form complete, V-shaped arc; T15 small, smooth extra bony protrusion cranial to normal right transverse process. Pig 8 eight articular process joint lesions: T10–11 left side, both sides of articulation OCD; T11–12 left side, one side of articulation, caudal T11 OCD; T13–14 left and right sides, caudal T13 articular processes short and articulate with base of T14 spinous process as T14 cranial articular process absent; T14–15 left side, both sides of articulation OCD and right side, one side of articulation caudal T14 OCD

**Fig. 4:**
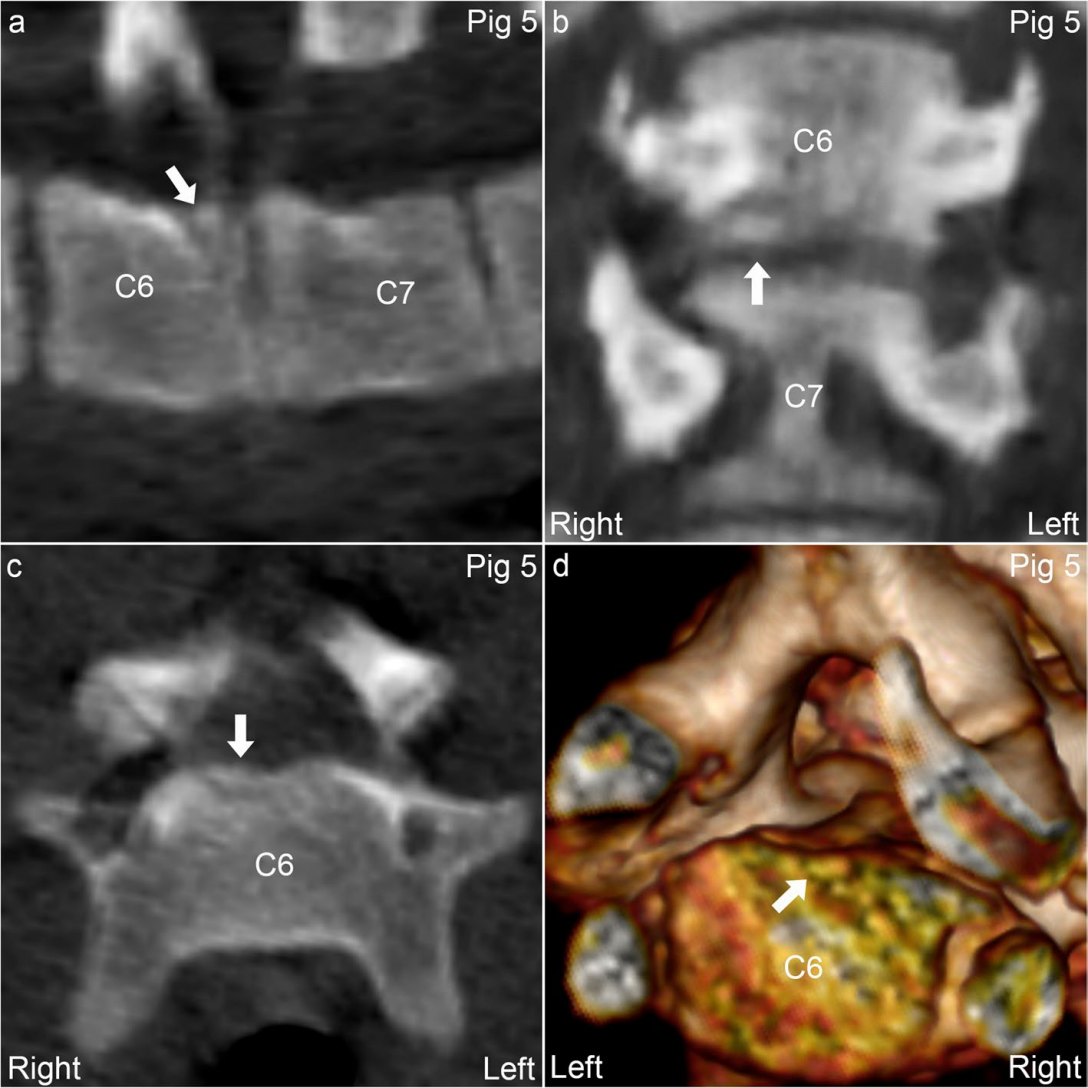
Osteochondrosis dissecans (OCD) in the neuro-central synchondrosis of pig 5. **a.** There is an OCD lesion (arrow) located towards the floor of the vertebral canal caudally in C6. **a.** The lesion (arrow) is located cranial to the caudal physis of C6, and **b-d.** near the midline, corresponding to the neuro-central synchondrosis. **a.** Sagittal plane. **b.** Dorsal plane. **c.** Transverse plane. **d.** 3D model, caudal and slightly right-to-left oblique view

**Fig. 5:**
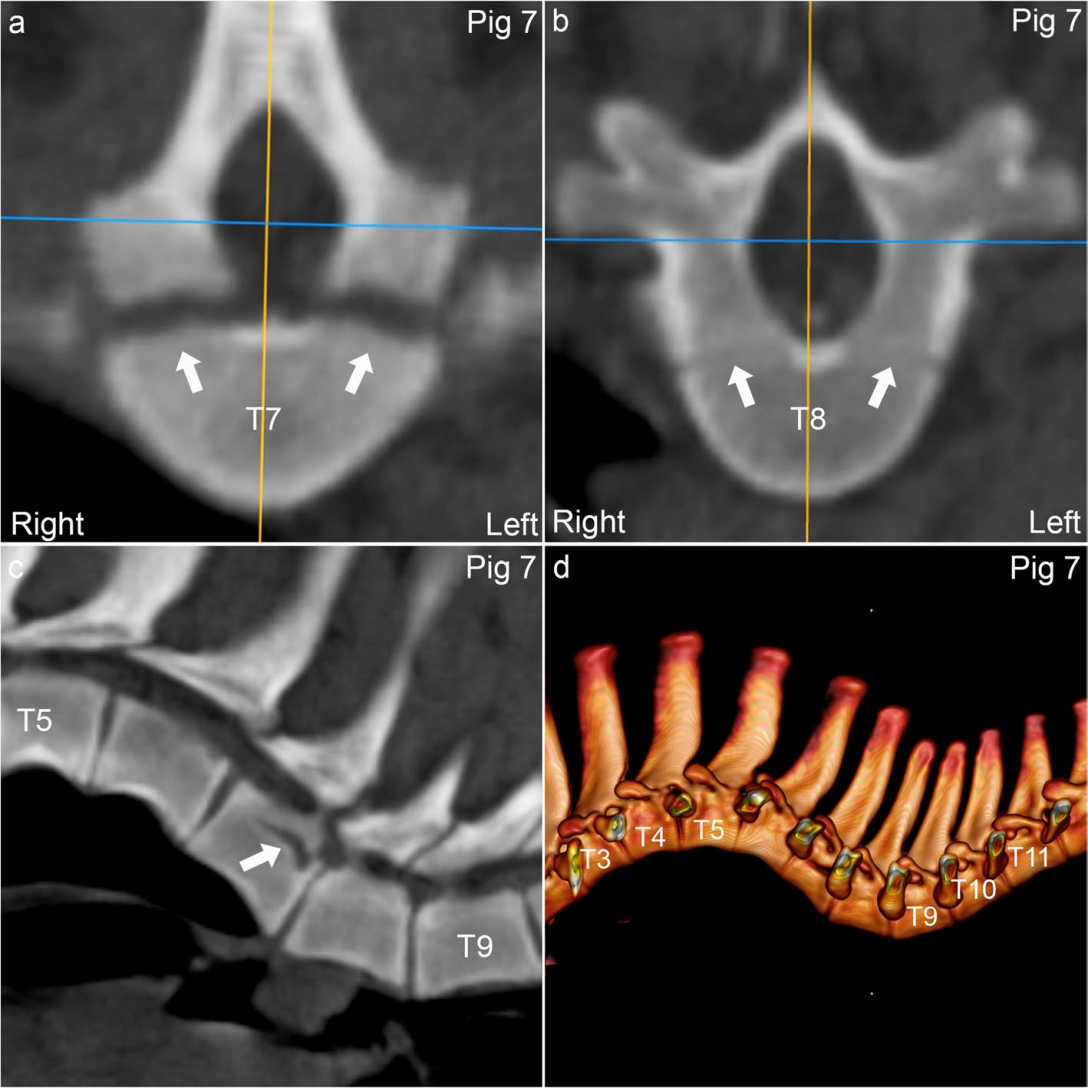
Pathological Type I Salter-Harris fracture of the neuro-central synchondrosis of pig 7*. **a.** The neuro-central synchondroses (arrows) caudally in T7 were 2–3 times normal thickness. **b.** Image of the neuro-central synchondroses (arrows) of T8 for comparison. **c.** The increased thickness (arrow) was interpreted as a pathological, Type I Salter-Harris fracture of the neuro-central synchondrosis in T7 secondary to the visible ventral wedging of T6 and dorsal wedging of T8. **d.** The wedging is associated with marked kyphosis and lordosis. **a-b.** Transverse plane. **c.** Sagittal plane. **c.** 3D model, left view

Pig 5 had lesions at C6–7 without wedging. In the body of C6, there was an OCD lesion towards the floor of the vertebral canal, originating in the growth cartilage of the neuro-central synchondrosis (Fig. 4), and a cyst in the right transverse process. The caudal metaphyseal growth cartilage of C6 was thinner and more heterogeneous in density than neighbouring growth plates, potentially compatible with radiological “physitis”, and the ventral parts of C6 and C7 were connected by a small, bony bridge/spondylosis. In pig 7*, the neuro-central synchondroses increased symmetrically from normal thickness cranially to 2–3 times normal thickness caudally within T7 (Fig. 5a-c). This could represent disturbed ossification, but as the adjacent vertebrae were prominently wedged (Fig. 5c-d), it was considered more likely that the lesion reflected pathological, Type I Salter-Harris fracture of the synchondroses in T7, secondary to the adjacent wedging.

**Fig. 6:**
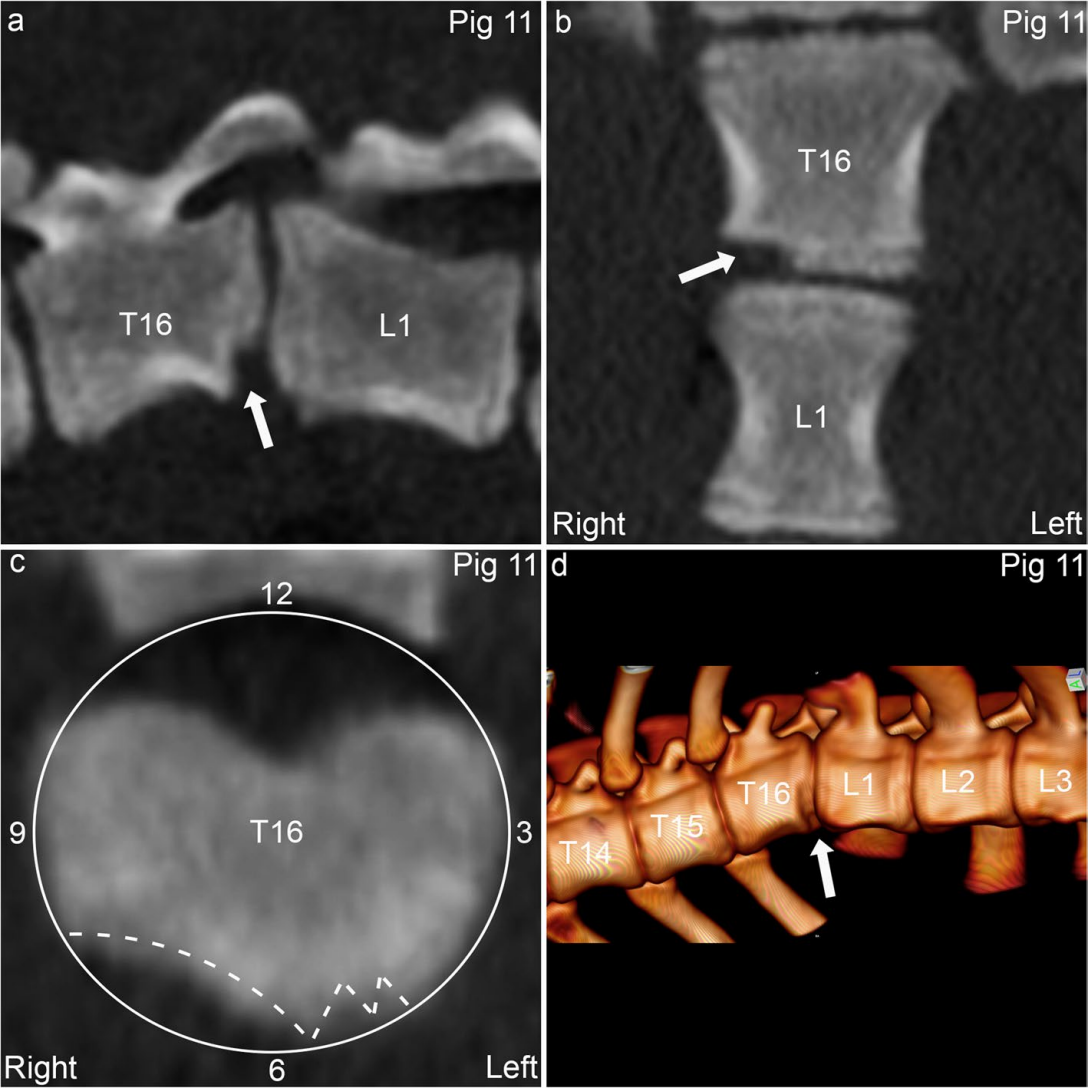
Ventral vertebral body osteochondrosis and kyphosis in pig 11. **a.** There is an osteochondrosis lesion (arrow) affecting both the physis and the epiphysis caudo-ventrally in T16. **b.** The osteochondrosis lesion (arrow) is located to the right of the midline. **c.** The lesion (dashed lines) is located between 5 and 8 o’clock on a clockface (circle and 3, 6, 9 and 12 indicators) superimposed on the vertebral body in the transverse plane. **d.** There is mild kyphosis centred at T16 with the osteochondrosis lesion (arrow). **a.** Sagittal plane. **b.** Dorsal plane. **c.** Transverse plane. **d.** 3D model, left and slightly left-ventral-to-right-dorsal oblique view

**Fig. 7:**
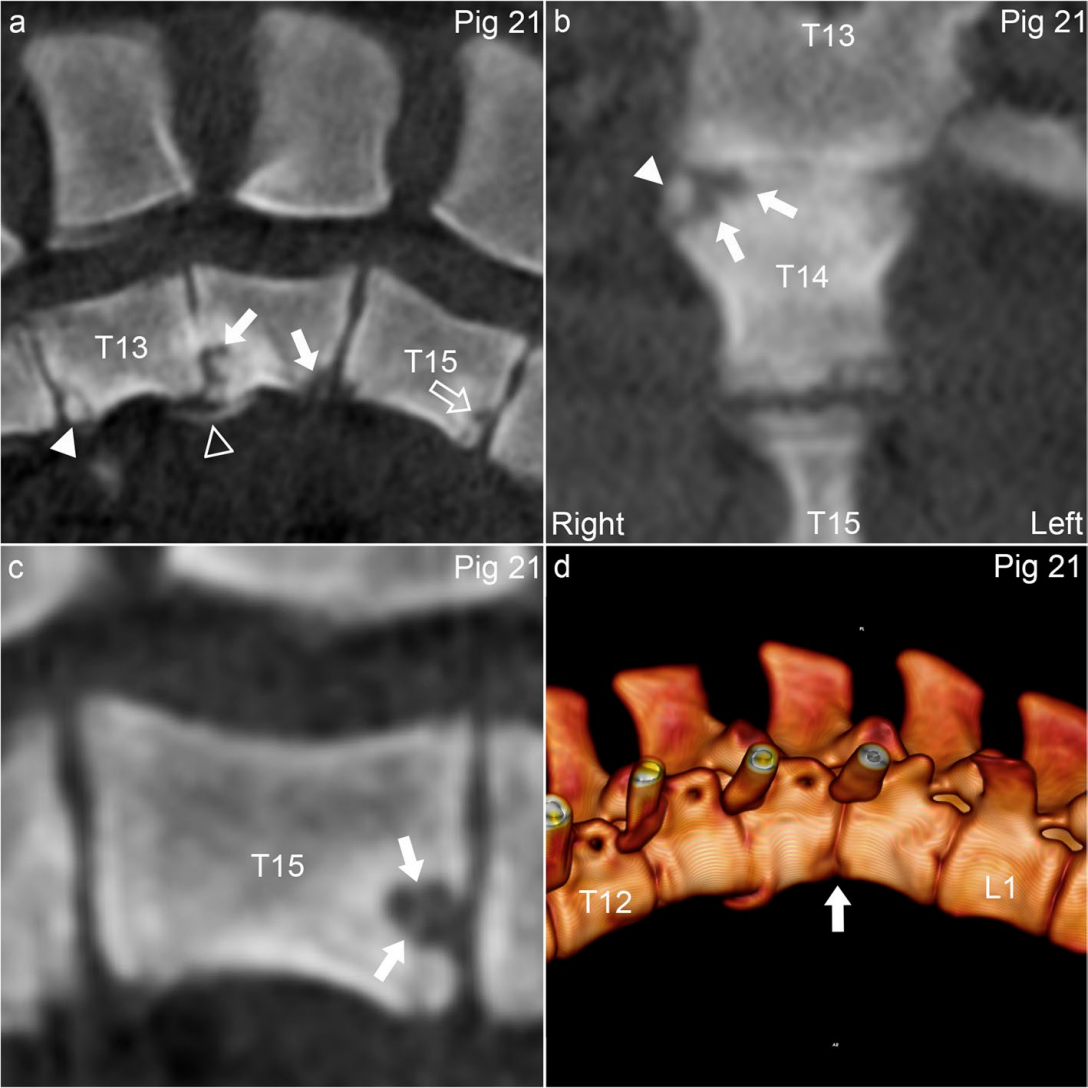
Multiple vertebral body osteochondrosis lesions, osteochondrosis dissecans (OCD), cyst, spondylosis, wedging and kyphosis in pig 21. **a.** There are osteochondrosis lesions (arrows) cranio- and caudo-ventrally in T14, an OCD lesion (arrowhead) cranio-ventrally in T13 and a cyst (open arrow; for non-tangential section, see **c.**) caudally in T15, all of which are also ventral wedge vertebrae. A smooth, bony spur (open arrowhead) extends from the cranio-ventral aspect of T14 towards the caudo-ventral aspect of T13, interpreted as bone bridge formation/spondylosis. **b.** The osteochondrosis lesion (arrows) cranially in T14 is multi-lobulated and located to the right of the midline. The mineral opacity (arrowhead) superficial to the lesion is cut tangentially here and in **a.** and was connected to the parent bone in other planes of section, interpreted as reparative ossification. **c.** Para-sagittal slice through the centre of the cyst (arrows) caudally in T15. **d.** There is mild kyphosis centred at the T14-T15 junction (arrow). **a-c.** Sagittal plane. **b.** Dorsal plane. **d.** 3D model, left view

Pig 11 had a single, multi-lobulated osteochondrosis lesion affecting both the physis and the epiphyseal growth cartilage caudally in T16 (Fig. 6), associated with ventral and right wedging, kyphosis and lordosis (Table 2). Pig 21 had multi-lobulated osteochondrosis lesions cranio- and caudo-ventrally in T14 (Fig. 7a-b), an OCD lesion cranio-ventrally in T13 (Fig. 7a) and a cyst caudally in T15 (Fig. 7a, c), all of which were also ventral wedge vertebrae associated with kyphosis (Fig. 7d; Table 2). A smooth, bony spur extended from the cranio-ventral aspect of T14 towards the caudo-ventral aspect of T13, interpreted as bone bridge formation/spondylosis (Fig. 7a, d).

**Fig. 8:**
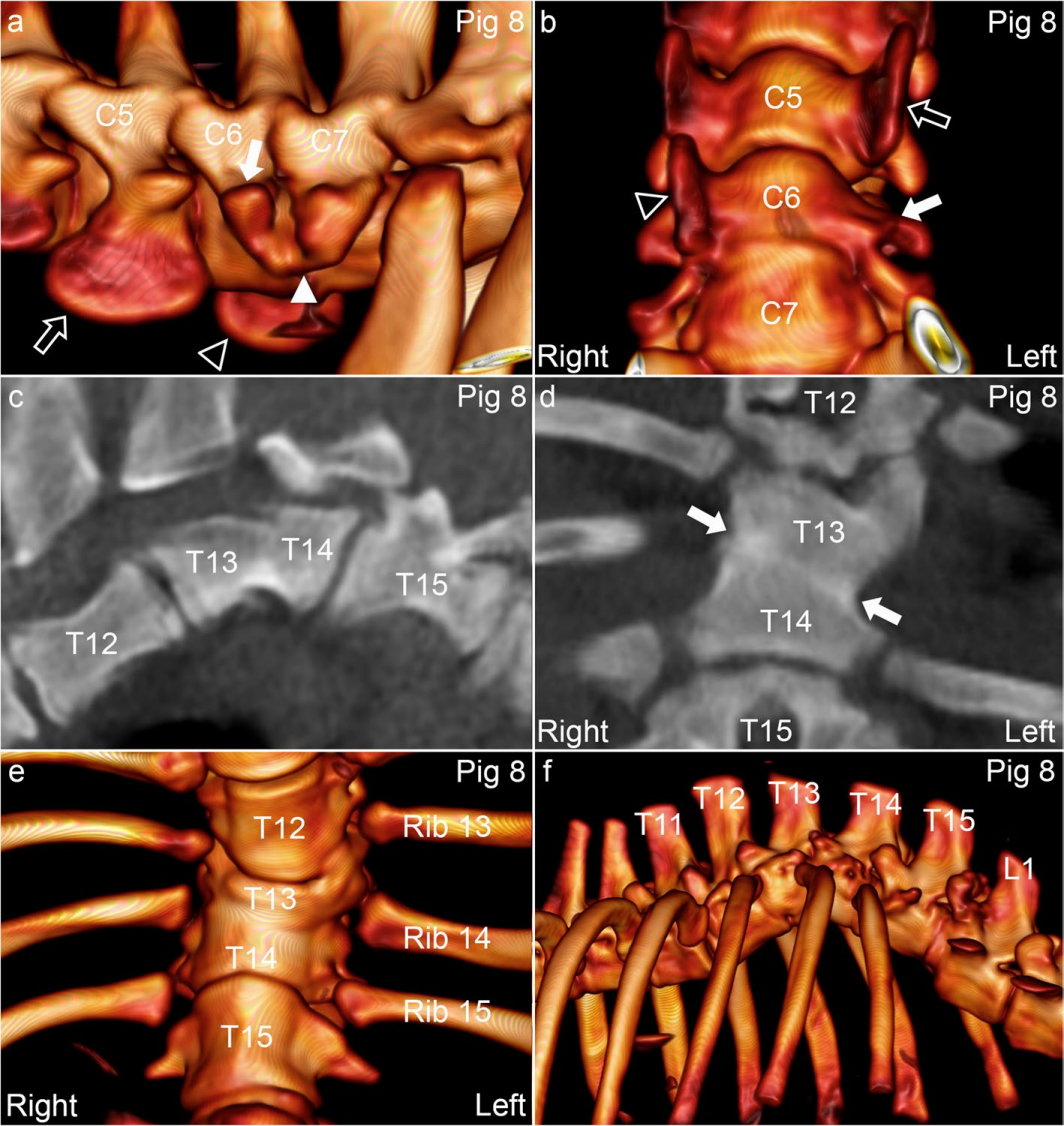
Cervical transverse process “transposition”, block vertebra and kyphosis in pig 8. **a-b.** The left transverse process of C6 (arrow) is small and appears to be fused (arrowhead) to the transverse process of C7. The left transverse process of C5 (open arrow) is large and matches the right transverse process of C6 (open arrowhead) in size, compatible with malformation/“transposition” of the left transverse process of C6 to C5. **c.** The intervertebral disc and secondary ossification centres are absent between the vertebral bodies of T13–14, which are fused into a ventrally wedged block vertebra. **d.** An oblique, hyperdense line (between arrows) marks the junction between the primary ossification centres, and T14 is located slightly to the right of T13, **d. e.** causing the spinal axis to shift to the right from T14 caudally. **e.** The block vertebra articulates with rib pairs 13, 14 and 15. **f.** There is marked kyphosis centred at the T13–14 block vertebra. **a, f.** 3D models, left views. **b, e.** 3D models, ventral views. **c.** Sagittal plane. **d.** Dorsal plane

Pig 8 had a small transverse process on the left side of C6, apparently fused to the transverse process of C7, whereas the left transverse process of C5 was large and matched the right transverse process of C6 in size, compatible with malformation/“transposition” of the left transverse process of C6 to C5 (Fig. 8a-b). The disc and secondary ossification centres were absent between T13–14, which were fused into a ventrally wedged block vertebra (Fig. 8c-f). A somewhat oblique, hyperdense line marked the junction between the primary ossification centres, and T14 was located slightly to the right of T13, causing the spinal axis to shift to the right from T14 caudally (Fig. 8d-e). The block vertebra articulated with three pairs of ribs (Fig. 8e-f), and there were multiple changes in the transverse processes of T11–15, including absence, new bone formation and size and shape abnormalities (listed in the legend of Table 5). There were remnants of very small osteochondrosis lesions in T13-L1, listed in Table 5.

### Relationship between focal lesions and wedge vertebrae

There were 10 pigs with wedge vertebrae, and of these, pigs 2*, 12* and 18* had focal lesions elsewhere in the spine (Table 3), but not in the wedge vertebrae (Table 2).

Pigs 11 and 21 had both: osteochondrosis lesions ventrally in intervertebral joints and: ventral wedging (Table 2). This included the mismatch that there were left lesions, without left-sided wedging in T13 and T15 of pig 21. Similar co-localization was apparent between ventral, left-sided intervertebral osteochondrosis and ventral wedging, fusion and right-shifting of T13–14 in pig 8, but these ventrally wedged vertebrae also had small lesions in the dorsally located articular process joints (Table 2).

Conversely, pigs 1, 15* and 34 had both: osteochondrosis lesions in dorsal articular process and rib joints and: dorsal wedging (Table 2). Of these, pig 1 had symmetric lesions and wedging, whereas pig 15* had left-sided lesions without left wedging and pig 34 had right-sided lesions without right wedging. Finally, pig 7* had both a neuro-central synchondrosis lesion and dorsal wedging of T7, but it is not known whether the lesion was a consequence, or the cause of the wedging.

In sum, there were 21 wedge vertebrae and on 10 occasions, they contained no lesions, whereas on six occasions, ventral wedge vertebrae contained lesions ventrally in intervertebral joints and on five occasions, dorsal wedge vertebrae contained lesions in the dorsally located synchondrosis, articular process or rib joints.

## Discussion

The main finding was that 73% of the pigs with poor back conformation had normal spines, whereas 27% of the pigs had abnormal spines and all of them had wedge vertebrae.

### Vertebral number and transitional vertebrae

The number of vertebrae varied, but the current study contained too few pigs to make any inferences about the relationship between vertebral number and abnormal curvature. In Holl et al.’s [8] heritability study with > 1500 pigs, there was no correlation between kyphosis and number of vertebrae or back length. Transitional vertebrae are rarely reported, so it is difficult to know what to compare the current 22% prevalence of transitional vertebrae to. Thoraco-lumbar transitional vertebrae were not associated with abnormal curvature in the current examined pigs, but in humans, lumbo-sacral transitional vertebrae have been associated with lower back pain [25], and this is worth remembering when considering the relationship between conformation and spine abnormalities.

### Pigs with abnormal spine curvature and abnormal vertebrae

The fact that there were many more pigs with poor conformation than with abnormal spines was anticipated [3, 8] and is readily explainable. Some poor conformation scores may represent observer error and theoretically, it could be good to develop a method for automated, objective conformation scoring. There may have been a genuine problem within the spine, affecting hypodense soft tissues rather than bone including the potential pain from transitional vertebrae mentioned above [25]. Poor conformation could also represent a postural response to limb pain [2, 3], and all current examined pigs had elbow or stifle osteochondrosis (Supplemental Table 3), a prevalence that is within the reported range for the included breeds [26].

The mismatch between conformation scores and spine abnormality means that there is a risk of classifying poor conformation as being due to spine abnormality, when it in fact is due to something else, for example limb osteochondrosis [2, 3]. Misclassification may be of little practical consequence, as selection against poor back conformation will still give breeding progress, just against limb osteochondrosis rather than spine abnormality. In genetic studies, misclassification can be a serious error, especially if it leads to the conclusion that a chromosome region is associated with one disease, when it is really associated with another. The best way to avoid such error would be to classify the spine (as well as the limbs) directly in CT scans, rather than indirectly via inference from back conformation.

### Focal lesions in articular process and rib joints

It was somewhat overwhelming, but not entirely unexpected to detect as many as 875 lesions, 98.5% of which represented stages of osteochondrosis [24], in the articular process and rib joints of every pig. This agrees with Reiland detecting osteochondrosis lesions in “synovial intervertebral joints” in 80% of his pigs, particularly in the lumbar segment [19]. It is difficult to know if rib joints were included in Reiland’s synovial joint category [19], but if they were not, this could explain the even higher prevalence in the current pigs that were also pre-selected for having poor back conformation. The current observed lesions were located towards the epiphyseal ends of articular and transverse processes and in limbs, this would correspond to failure of anatomical end arteries during incorporation of the mid-portion of cartilage canal vessels into the advancing ossification front during growth [27, 28]. The working hypothesis is that vessels are unable to withstand the micro-mechanical forces in regions where they traverse the chondro-osseous junction after incorporation into bone [27, 28]. Based on existing theories about limb osteochondrosis, it is possible to generate some hypothetical explanations for characteristics of the current observed articular process and rib joint lesions:

- Multiple affected vertebrae and symmetrically affected joints: equine limb osteochondrosis affects more than one joint simultaneously in > 50% of cases, and multiple affected joints are more often symmetric than asymmetric [29, 30]. This is believed to be because the blood supply is configured, develops and is present for the same period of time in symmetric joints, meaning they have similar windows of vulnerability to vascular failure [31]. Conversely, asymmetric lesions are believed to be a result of the balance between the number of lesions that are *initiated*, and the proportion that *resolves* [32], discussed further below.
- Predilection segments and sites: in the equine hock [28], stifle [33] and fetlock joints [34], predilection sites for osteochondrosis tended to correspond to sites where the temporary blood supply regressed last, i.e., sites that had the longest windows of vulnerability to vascular failure.
- Different proportion of osteochondrosis lesion stages between segments: in the pathogenesis, osteochondrosis and cystic lesions represent stages of increasing duration [15, 24, 35]. Growth closes at a younger age in distal, compared to proximal limb joints, meaning there can be early lesions in proximal limb joints at an age when distal limb lesions are chronic. Higher proportion of osteochondrosis may therefore indicate that growth closes at an older age in the cervical, compared to the thoracic and lumbar segments [10].
- The caudal side was affected more frequently than the cranial side in the cervical segment, whereas the reverse was true for the thoracic and lumbar segments. It would be interesting to discover whether the blood supply is different in such a way that it explains why more lesions may be *initiated* in cranial, compared to caudal articular processes. However, Reiland [19] observed more severe lesions in caudal than cranial lumbar articular processes, and one of the most likely explanations for this discrepancy is that selection has resulted in the 2015-generation of pigs having longer backs than the 1978-generation of pigs, making it necessary to consider the influence of biomechanical force. In this context, it is important to be aware that the extra-cellular matrix [36] and subchondral bone [37] were intact at sites of vascular failure when examined using techniques capable of detecting disruption. This means that to explain *initiation* of (non-septic) osteochondrosis [36, 37], biomechanical force must be able to disrupt just cartilage canal vessels, whilst leaving the surrounding tissues intact. The natural curvatures of the spine may involve foci of increased force; cranial and caudal articular processes have slightly different shape; and the cervical segment is oriented slightly more vertical than the thoracic and lumbar segments; and these are just some suggestions for how biomechanical forces may be different in such a way that it explains why fewer lesions may be able to resolve at osteochondrosis predilection sites within the spine [32]. The ultimate way to start investigating some of the above proposed hypotheses would be to conduct systematic studies of the development of the blood supply to growth cartilage at predilection and control sites, as previously done in the spine of humans [11, 12] and rabbits [13, 14].

### Focal lesions in parts of vertebrae other than articular process and rib joints

Initially, the defect in T7 of pig 7* resembled a fracture line (Fig. 5a, c), but closer inspection revealed that it was symmetric and that there were similar, thinner lines in other vertebrae (Fig. 5b), prompting renewed literature search and discovery of the neuro-central synchondrosis [10, 38]. The synchondrosis has a blood supply [11, 13], and it may therefore be vulnerable to vascular failure and osteochondrosis. Strictly speaking, the lesion in pig 7* consisted just of gradual widening of the synchondrosis, and definitive diagnosis of whether the widening was due to delayed ossification or fracture would have required histological validation. However, the OCD lesion in C6 of pig 5 (Fig. 4) supports that primary osteochondrosis does indeed occur within the neuro-central synchondrosis of pigs. Unilateral synchondrosis tethering reliably produces scoliosis [22, 38, 39], and different tethering techniques have variably been reported to result in rotation, stenosis and lordosis [22, 38, 39], thus the synchondrosis clearly warrants further investigation.

The osteochondrosis lesions observed mainly ventrally in the current examined pigs (Figs. 6, 7 and 8) appear to be identical to the lesions that were histologically validated by Reiland [19], Nielsen et al. [1] and Corradi et al. [5] (own example in Supplemental Fig. 1). After vertebral osteochondrosis, the second-largest group of changes in the current pigs was that major parts of vertebrae were absent: the transverse process of C6, the secondary epiphyseal ossification centres of the T13–14 block vertebra (Fig. 8) and several transverse processes from T11-T15 in pig 8. There are three main options for why parts of the spine may be absent: they may have failed to separate properly during embryonic segmentation [40, 41], they may have segmented, but ossification centres may have failed to form [1], or ossification centres may have formed, but been destroyed, for example through discospondylitis [42]. Vessels are present before and essential to formation of ossification centres [11–14, 43], thus vascular failure may theoretically cause centres to fail to form altogether. However, in limb osteochondrosis, vascular failure occurs during vessel incorporation into *secondary* ossification centres [18, 27, 28], and vessels cannot possibly fail during incorporation into secondary centres that never form as in T13–14 of pig 8 and Nielsen et al. [1]. Instead, vessels may fail during incorporation into the advancing ossification front of the *primary* ossification centre, made plausible by such incorporation being visible in the figures of Amato et al. [13] and Skawina et al. [11]. Failure of vessels during incorporation into primary ossification centres could then explain why secondary ossification centres were absent in pig 8.

### Relationship between focal lesions and wedge vertebrae

In this cross-sectional study, we were obliged to describe the lesions that were present in the wedged vertebrae at the time of the CT scanning. Reiland [19] documented early physeal osteochondrosis lesions in 4–5-month-old pigs and angular limb deformity in 8–9-month-old pigs, by which age the initial osteochondrosis lesions had resolved [21]. When pigs are used as scoliosis models, timing varies with breed, age, site and technique but it generally takes ≥4 weeks for angulation to manifest [22, 38, 39]. According to this, wedge vertebrae without lesions should be interpreted to be due to osteochondrosis lesions that were present ≥4 weeks ago, whereas lesions present in wedge vertebrae could cause further wedging over the ≥4 weeks after the CT-scanning [22, 38, 39]. Ultimately, the question of whether ventral vertebral osteochondrosis causes wedging and juvenile kyphosis should be answered through longitudinal monitoring of pigs with spontaneous lesions [21]. In the interim, the following evidence firmly supports that ventral osteochondrosis causes kyphosis:

- Ventral osteochondrosis and ventral wedging were centred at the same sites [1, 5, 19].
- Osteochondrosis is the commonest developmental disease, it is defined as a focal delay in endochondral ossification [3, 19], and delayed ossification is one mechanism by which one, e.g., the ventral side of a vertebra may end up shorter than the dorsal side.
- In studies where pigs are used as model animals, tethering of the ventral aspect of vertebral bodies results in kyphosis [44].

Shortening of the dorsal relative to the ventral side may be due to delayed ossification within structures in the dorsal half of vertebrae like the articular process or rib joints. Ribs have been manipulated, including tethering of adjacent ribs in lordotic-type scoliosis models [22], but osteoarthritis with bridging osteophytes was only detected in single joints in the current observed naturally occurring disease and such single-joint “tethering” was not associated with wedging or scoliosis at the time of sampling. We hesitate to rule out the possibility that articular process and rib joint osteochondrosis may result in vertebral wedging in other pigs, or other species entirely. However, in the current examined pigs, articular process and rib joint osteochondrosis lesions were present so frequently (> 800 times) in normal vertebrae that they may just represent incidental findings when they occasionally also were present in wedge vertebrae. Conversely, unilateral tethering of the neuro-central synchondrosis reliably produces scoliosis [22, 39], so the role of this structure in lateral (and dorsal) wedging definitely warrants further investigation.

### Confounding errors

Medications were carefully registered to identify infections and avoid confounding error (Supplemental Table 1). The lesions in pigs 7*, 15* and 18* may have been due to either aseptic or septic vascular failure [16, 17], whereas the lesions in the remaining seven pigs that did not receive any medications were more likely to have been aseptic, i.e. presumed heritably predisposed. Initial susceptibility to septic failure may be limited to specific ages because it has been associated with discontinuities present during active in-growth [45] or physiological regression [46]. Thus aseptic, presumed heritably predisposed vascular failure may be responsible for the baseline 2.5–11.4% prevalence of humpback [4], whereas the outbreaks with > 30% mortality [5, 6] and concurrent pneumonia [2, 4] are potentially compatible with septic vascular failure [16, 17] during the physiological regression susceptibility window [46].

Manipulating vitamin D, calcium and phosphorus in the diet of pregnant sows and/or piglets induces outbreaks of 20–30% juvenile kyphosis [47, 48], and it would be interesting to know whether this occurs via any effect on blood supply [49], but the diet of the current pigs was balanced, verified annually and did not cause confounding error.

### Limitations

Conformation scoring [50] was incomplete in the sense that it did not include all three planes, but the scoring was only used to select a relevant sample from the > 20,000 available pigs, and any comparisons of curvature, wedging and lesions were done within the CT scans, not to the conformation scores. The study did not include a control group with optimal conformation scores, mainly because pigs with normal backs can have abnormal spines [8].

## Conclusions

Computed tomography was suited for identification of wedge vertebrae, and kyphosis was due to ventral wedge vertebrae compatible with heritably predisposed vertebral body osteochondrosis. Articular process and rib joint osteochondrosis may represent incidental findings in wedge vertebrae. The role of the neuro-central synchondrosis in the pathogenesis of vertebral wedging warrants further investigation.

## Methods

### Study design and pigs

The study was conducted by retrospectively extracting data from the boar-testing database of the Norwegian pig breeders’ association, Norsvin SA (www.norsvin.no) from 2008 to the end of 2015. Pig owners consented to boars being tested, and to test data being used for research. All pigs were kept in accordance with the national legislation (Animal Welfare Act LOV-2021-06-18-134; Regulation for the keeping of pigs in Norway FOR-2020-06-10). The study was approved by the institution ethical committee (Ref. 14/04723–68).

Inclusion criteria for the boar test were that every year, 3500 boars were selected for testing from nucleus herds of purebred Landrace or Duroc pigs based on pedigree analysis [23]. The test period started at ~ 25 kg live weight. Pigs that developed disease that did not respond to treatment during the test were excluded. Pigs were CT-scanned at 100 kg live weight until 1.3.2012, and at 120 kg thereafter (market-regulated). Landrace boars were a mean of 146 days at 100 kg [26] and 165 days at 120 kg, and Duroc boars were approximately 10 days older at the same weights [51]. Conformation scoring was carried out ≤10 days after CT-scanning.

Inclusion criteria for the current study were that a CT scan and a conformation score had to be available from the pig. The conformation scores were used to select a relevant sample from the > 20,000 available pigs, and using inclusion scores of severe for dipped back, and moderate or severe for humpback (see below) resulted in a sensible sample size.

### Historical methods: CT-scanning and conformation scoring

The CT-scanning [23] and conformation scoring [50] have been described before, but briefly: boars were sedated and positioned in sternal recumbency with free limb position in a gurney that was curved to fit inside the CT gantry. A latero-lateral scout image was obtained and collimated to acquire ~ 1100 transverse images in ~ 90 s from the snout to the tail of each pig. Scan parameters were optimised for lean meat and fat quantification [23] and slice thickness was 0.625–1.25 mm.

Conformation scoring was carried out by experienced technicians who assessed 32 traits in ~ 5 min per pig. Dipped back was assessed throughout the study period, whereas humpback was assessed from 1.3.2012 onwards and lateral deviation was not assessed. Both dipped and humpback were assessed on a scale of normal, mild, moderate or severe [50].

### Evaluation of the CT scans

The CT scans were imported into a software package (Horos v. 3.3.6; www.horosproject.org) and read by a veterinary radiologist with 14 years’ experience. Reproducibility was informally tested by reading 148 C7-T1 articulations two times > 1 month apart, with agreement on 136/148 (92%) occasions. Disagreement was handled by reviewing both readings a third time and keeping all lesions agreed upon at that time.

### Parameters observed

Vertebrae were counted according to convention [52], including identification of the anticlinal vertebra towards which the dorsal spinous processes of all other vertebrae incline. Transitional vertebrae with characteristics of both the cranially and caudally adjacent segments (Fig. 2) were registered and counted according to convention [52].

### Spine curvature

Dorso-ventral curvature was assessed in the sagittal reconstructed slices and judged in the median plane. If possible, the entire spine was viewed in a single, median image, but if positioning or deformity precluded this, shorter portions of the spine were judged at a time and the viewing plane was adjusted to always be as close to the median plane as possible.

Lateral curvature was assessed in the dorsal reconstructed slices and judged both in 3D models and at mid-height of the vertebral bodies. To compensate for the natural curvature of the different segments, the curved reconstruction function of the software was used to always align the viewing plane perpendicular to the mid-height dorsal plane of as many adjacent vertebrae as possible.

If the spine was straight or gradually, smoothly and evenly curved, it was categorised as having normal curvature. If curvature deviated sharply over a limited number, approximately 1–5 vertebrae, the spine was categorised as having abnormal curvature, further specified as kyphosis, lordosis and left or right scoliosis.

### Vertebral shape

Vertebrae in spines with normal curvature were considered to have normal shape and used as comparisons. Vertebrae where the ventral contour was excessively shorter than the dorsal contour were referred to as ventral wedge vertebrae, and when the dorsal contour was shorter than the ventral contour, this was referred to as dorsal wedging. Vertebrae where the left side was shorter than the right side were referred to as left wedge vertebrae and vice versa.

### Focal lesions

All vertebrae and articulations starting with the atlanto-occipital joint up to and including the lumbo-sacral junction were evaluated in three orthogonal planes, including all intervertebral disc joints, articular process joints (zygapophyseal or facet joints), rib head joints (costo-vertebral joints) and rib tubercle joints (costo-transverse joints).

Five categories of lesions were recorded:

- Osteochondrosis lesions: focal, sharply demarcated, uniformly hypodense, single- or multi-lobulated (“stair-step”) defects in the ossification front (Fig. 3a) [24]. Bone densities protruding adjacent to osteochondrosis lesions that were visibly connected to the parent bone were interpreted as reparative ossification.
- OCD: mineral, hyperdense bodies separate from and present superficially or laterally adjacent to osteochondrosis defects were interpreted as OCD lesions (Fig. 3b) [28, 35].
- Cyst: roughly spherical defects located immediately deep to the ossification front and surrounded by bone on most of their periphery (Fig. 3c) [24, 35, 53]. When both cysts and osteochondrosis were present, lesions were recorded as cysts by default.
- Osteoarthritis: joints with reduced or irregular joint space, subchondral bone sclerosis and periarticular osteophytes were categorised as osteoarthritic (Fig. 3d-e).
- Other lesions: lesions that did not fit into any of the above categories were described on an individual basis.

The location of each articular process joint lesion was recorded in terms of left or right side and cranial, caudal or both sides of the articulation. For rib joint lesions, location was recorded as caudal vertebral, cranial vertebral, rib or ≥ two sides. If the same articulation was affected on both the left and right sides of the pig, the lesions in those articulations were categorised as symmetric even if they were not an exact match in terms of side of the articulation or character.

The location of intervertebral disc joint changes was recorded by conventional viewing of the spine in the transverse plane with dorsal to the top and patient left to image right, and superimposing a clockface on the vertebral body such that 0–6 o’clock represented the left half and 3–9 o’clock represented the ventral half of vertebrae, etc. (Fig. 6c). Lesion location was then specified in terms of the times it spread from and to, and the time at which it was centred.

## Supplementary Information

**Additional file 1: Supplemental Table 1.** Treatments administered during the boar test.

**Additional file 2: Supplemental Table 2.** Distribution of the 875 articular process and rib joint lesions among the 30 articulation levels raw data.

**Additional file 3: Supplemental Table 3.** Elbow and stifle osteochondrosis in the study population.

**Additional file 4: Supplemental Figure 1.** Ventral vertebral lesion represents ischaemic chondronecrosis in a 12 kg piglet. a. The secondary ossification centres (2°) are absent caudo-ventrally in T8 and cranio-ventrally in T9. There is a lesion centred at the junction between T8-T9; higher magnification of the tissue inside the dashed box is shown in b. b. The lesion consists of necrotic cartilage canal vessels (asterisks), surrounded by necrotic chondrocytes (within dashed lines), representing ischaemic chondronecrosis identical to limb osteochondrosis. Viable chondrocytes (arrows) on the margin of the area of chondronecrosis are proliferating. a. The intervertebral disc appears to be absent, prompting the question of whether the lesion represents failure of the blood supply to growth cartilage, to the intervertebral disc, or both. a-b. Para-sagittal histological section from T8-T9 of a 12 kg mixed-breed piglet; a. 10x; b. 100x magnification, haematoxylin and eosin.

## Data Availability

The datasets used and analysed in the study are available from the corresponding author on reasonable request.

## Abbreviations

CT: Computed tomography
OCD: Osteochondrosis dissecans
C: Cervical
T: Thoracic
L: Lumbar
S: Sacral
P: Metaphyseal growth plate or physis
EGC: Epiphyseal growth cartilage
S: Synchondrosis
PFZ: Proliferative zone of growth cartilage
