# Supplementary material for: Osteochondrosis and other lesions in all intervertebral, articular process and rib joints from occiput to sacrum in pigs with poor back conformation, and relationship to juvenile kyphosis
Source: BMC Vet Res. 2022 Jan 18;18:44. doi: 10.1186/s12917-021-03091-6 (PMC8764802; doi:10.1186/s12917-021-03091-6)
Supplement: Supplementary file 1 — Additional file 1: Supplemental Table 1. Treatments administered during the boar test. [file 12917_2021_3091_MOESM1_ESM.docx]

**Supplemental table 1**. Treatments administered during the boar test

| **Pig** | **Days before CT scan** | **Indication** | **Medication** |
| --- | --- | --- | --- |
| 2 | 29 days | Skeletal disease | Unknown |
| 6 | 89 days | Prevention of respiratory disease | Unknown |
| 7 | 78 days | Gastro-intestinal disease | Unknown |
| 7 | 67 days | Anorexia | Unknown |
| 10 | 51 days | Infection other than respiratory infection | Unknown |
| 10 | 40 days | Prevention of respiratory disease | Unknown |
| 10 | 27 days | Respiratory disease | Unknown |
| 12 | 25 days | Unknown | Selenium/vitamin E |
| 15 | 26 days | Respiratory disease | Penicillin  Meloxicam |
| 16 | 145 days | Musculo-skeletal disease | Unknown |
| 18 | 104 days | Joint disease | Penicillin  Dexamethasone  Selenium/vitamin E |
| 18 | 74 days | Respiratory disease | Penicillin  Meloxicam |
| 18 | 38 days | Respiratory disease | Amoxicillin  Dexamethasone  Vitamin B |
| 18 | 15 days | Joint disease | Dexamethasone  Selenium/vitamin E |
| 18 | 13 days | Joint disease | Amoxicillin  Meloxicam |
| 22 | 68 days | Skin infection | Amoxicillin |
| 22 | 14 days | Joint disease | Penicillin  Meloxicam |
| 23 | 36 days | Respiratory disease | Penicillin  Meloxicam |
| 25 | 88 days | Respiratory disease | Penicillin  Meloxicam |
| 26 | 43 days | Joint disease | Dexamethasone  Selenium/vitamin E |
| 27 | 11 days after CT scan | Respiratory disease | Penicillin  Meloxicam |
| 28 | 31 days | Respiratory disease | Penicillin  Meloxicam |
| 28 | 28 days | Respiratory disease | Penicillin  Meloxicam |
| 28 | 26 days | Respiratory disease | Tiamulin  Dexamethasone  Vitamin B |
| 29 | 69 days | Respiratory disease | Penicillin  Meloxicam |
| 29 | 76 days | Gastro-intestinal disease | Trimethoprim  Vitamin B |
| 29 | 34 days | Tail-biting | Penicillin  Dexamethasone |
| 30 | 45 days | Respiratory disease | Penicillin  Meloxicam |
| 30 | 20 days | Respiratory disease | Penicillin  Meloxicam |
| 30 | 11 days | Respiratory disease | Penicillin  Meloxicam |
| 33 | 47 days | Respiratory disease | Penicillin  Meloxicam |
| 37 | 21 days | Tail-biting | Penicillin  Meloxicam |

Please note: all pigs were vaccinated against *Actinobacillus pleuropneumoniae* and received azaperone sedation for the CT scans.
