# Supplementary material for: Osteochondrosis and other lesions in all intervertebral, articular process and rib joints from occiput to sacrum in pigs with poor back conformation, and relationship to juvenile kyphosis
Source: BMC Vet Res. 2022 Jan 18;18:44. doi: 10.1186/s12917-021-03091-6 (PMC8764802; doi:10.1186/s12917-021-03091-6)
Supplement: Supplementary file 2 — Additional file 2: Supplemental Table 2. Distribution of the 875 articular process and rib joint lesions among the 30 articulation levels raw data. [file 12917_2021_3091_MOESM2_ESM.docx]

**Supplemental table 2.** Distribution of the 875 articular process and rib joint lesions among the 30 articulation levels raw data. These data are summarised in manuscript Table 4.

**a.** Cervical segment (All pigs: seven cervical vertebrae)

| **Articulation level** | **Sum left side** | **Left side^1^** | **Articular process** | **Right side^1^** | **Sum right side** | **Sum left and right** |
| --- | --- | --- | --- | --- | --- | --- |
|  |  | 1:0:1:0 = 2 | Occiput | 1:0:0:0 = 1 |  |  |
|  |  | 2:0:0:0 = 2 | C1 cranial AP^2^ | 1:1:0:0 = 2 |  |  |
| A-O^3^ joint | 4 | 0:0:0:0 = 0 | Both | 0:0:0:0 = 0 | 3 | 7 |
|  |  | 4:0:0:0 = 4 | C2 caudal AP | 1:0:0:0 = 1 |  |  |
|  |  | 1:0:1:0 = 2 | C3 cranial AP | 2:0:0:0 = 2 |  |  |
| C2-C3 | 12 | 4:2:0:0 = 6 | Both | 4:2:0:0 = 6 | 9 | 21 |
|  |  | 1:0:0:0 = 1 | C3 caudal AP | 2:0:0:0 = 2 |  |  |
|  |  | 1:1:0:0 = 2 | C4 cranial AP | 9:0:0:0 = 9 |  |  |
| C3-C4 | 9 | 6:0:0:0 = 6 | Both | 5:1:0:0 = 6 | 17 | 26 |
|  |  | 0:1:0:0 = 1 | C4 caudal AP | 4:0:0:0 = 4 |  |  |
|  |  | 2:1:0:0 = 3 | C5 cranial AP | 2:1:0:0 = 3 |  |  |
| C4-C5 | 12 | 4:4:0:0 = 8 | Both | 2:0:0:0 = 2 | 9 | 21 |
|  |  | 0:0:0:0 = 0 | C5 caudal AP | 0:0:0:0 = 0 |  |  |
|  |  | 2:1:0:0 = 3 | C6 cranial AP | 3:0:0:0 = 3 |  |  |
| C5-C6 | 5 | 1:1:0:0 = 2 | Both | 4:0:0:0 = 4 | 7 | 12 |
|  |  | 2:0:0:0 = 2 | C6 caudal AP | 3:1:0:0 = 4 |  |  |
|  |  | 0:0:1:0 = 1 | C7 cranial AP | 1:0:0:0 = 1 |  |  |
| C6-C7 | 5 | 2:0:0:0 = 2 | Both | 0:0:0:0 = 0 | 5 | 10 |
|  |  | 2:0:0:0 = 2 | C7 caudal AP | 2:0:0:0 = 2 |  |  |
|  |  | 1:0:0:0 = 1 | T1 cranial AP | 0:0:0:0 = 0 |  |  |
| C7-T1 | 11 | 6:0:0:2 = 8 | Both | 2:0:0:2 = 4 | 6 | 17 |
| Sum cervical segment | 58 | 42:11:3:2 | 90:17:3:4  26 cranial,  34 caudal,  54 both | 48:6:0:2 | 56 | 114 |

^1^Osteochondrosis: osteochondrosis dissecans: cyst: other = sum.^2^AP: Articular process. ^3^A-O: Atlanto-occipital.

b. Thoracic segment (Pigs had from 14-17 thoracic vertebrae, counted from cranial to caudal, T17: no lesions.)

| **Articulation level** | **Sum left** | **Rib tubercle^1^** | | **Rib head^1^** | | **Left side^1^** | **Articular process** | **Right side^1^** | **Rib head^1^** | | **Rib tubercle^1^** | | **Sum right** | **Sum left & right** |
| --- | --- | --- | --- | --- | --- | --- | --- | --- | --- | --- | --- | --- | --- | --- |
|  |  |  |  | 0:1:0:0 = 1 | Cd^2^ vb^3^ | See part a. | C7 cd AP^4^ | See part a. | Cd vb | 0:0:0:0 = 0 |  |  |  |  |
|  |  | 0:0:0:0 = 0 | Vb^3^ | 0:0:1:0 = 1 | Rib 1 |  |  |  | Rib 1 | 0:1:1:0 = 2 | Vb | 0:0:0:0 = 0 |  |  |
|  |  | 1:0:0:0 = 1 | Tub^5^ | 0:0:0:0 = 0 | Cr^6^ vb | 1:0:0:0 = 1 | T1 cr AP | 0:1:0:0 = 1 | Cr vb | 0:0:0:0 = 0 | Tub | 1:0:0:0 = 1 |  |  |
| C7-T1-Rib 1 | 17 | 0:2:0:0 = 2 | Both | 7:4:0:0 = 11 | All | 0:0:0:0 = 0 | Both | 0:0:0:0 = 0 | All | 7:2:0:0 = 9 | Both | 0:0:0:0 = 0 | 13 | 30 |
|  |  |  |  | 0:0:0:0 = 0 | Cd vb | 4:0:1:0 = 5 | T1 cd AP | 4:2:0:0 = 6 | Cd vb | 2:0:0:0 = 2 |  |  |  |  |
|  |  | 0:0:0:0 = 0 | Vb | 0:0:1:0 = 1 | Rib 2 |  |  |  | Rib 2 | 0:1:1:0 = 2 | Vb | 0:0:0:0 = 0 |  |  |
|  |  | 0:0:0:0 = 0 | Tub | 0:0:0:0 = 0 | Cr vb | 1:0:0:0 = 1 | T2 cr AP | 0:0:0:0 = 0 | Cr vb | 0:0:0:0 = 0 | Tub | 0:0:0:0 = 0 |  |  |
| T1-T2-Rib 2 | 17 | 0:2:0:0 = 2 | Both | 4:2:0:0 = 6 | All | 0:2:0:0 = 2 | Both | 2:0:0:0 = 2 | All | 5:0:0:0 = 5 | Both | 2:0:0:0 = 2 | 19 | 36 |
|  |  |  | Vb | 0:1:0:0 = 1 | Cd vb | 2:0:1:0 = 3 | T2 cd AP | 5:0:0:0 = 5 | Cd vb | 2:0:0:0 = 2 |  |  |  |  |
|  |  | 0:0:0:0 = 0 | Tub | 1:0:1:0 = 2 | Rib 3 |  |  |  | Rib 3 | 1:1:1:0 = 3 | Vb | 0:0:0:0 = 0 |  |  |
|  |  | 1:0:0:0 = 1 | Both | 0:0:0:0 = 0 | Cr vb | 0:1:0:0 = 1 | T3 cr AP | 0:0:0:0 = 0 | Cr vb | 0:0:0:0 = 0 | Tub | 0:0:0:0 = 0 |  |  |
| T2-T3-Rib 3 | 10 | 0:0:0:0 = 0 |  | 2:0:0:0 = 2 | All | 0:0:0:0 = 0 | Both | 2:0:0:0 = 2 | All | 5:2:0:0 = 7 | Both | 2:0:0:0 = 2 | 21 | 31 |
|  |  |  |  | 0:0:0:0 = 0 | Cd vb | 8:0:0:0 = 8 | T3 cd AP | 3:2:0:0 = 5 | Cd vb | 0:1:1:0 = 2 |  |  |  |  |
|  |  | 0:0:0:0 = 0 | Vb | 1:0:0:0 = 1 | Rib 4 |  |  |  | Rib 4 | 0:0:1:0 = 1 | Vb | 0:0:0:0 = 0 |  |  |
|  |  | 0:0:0:0 = 0 | Tub | 0:2:0:0 = 2 | Cr vb | 0:0:0:0 = 0 | T4 cr AP | 0:1:0:0 = 1 | Cr vb | 0:0:0:0 = 0 | Tub | 1:0:0:0 = 1 |  |  |
| T3-T4-Rib 4 | 18 | 0:0:0:0 = 0 | Both | 4:1:0:0 = 5 | All | 0:0:0:2 = 2 | Both | 2:0:0:0 = 2 | All | 4:3:0:0 = 7 | Both | 0:0:0:0 = 0 | 19 | 37 |
|  |  |  |  | 0:0:1:0 = 1 | Cd vb | 0:1:2:0 = 3 | T4 cd AP | 3:1:1:0 = 5 | Cd vb | 4:0:0:0 = 4 |  |  |  |  |
|  |  | 0:0:0:0 = 0 | Vb | 0:0:0:0 = 0 | Rib 5 |  |  |  | Rib 5 | 1:0:1:0 = 2 | Vb | 0:0:0:0 = 0 |  |  |
|  |  | 1:0:0:0 = 1 | Tub | 0:0:1:0 = 1 | Cr vb | 1:0:0:0 = 1 | T5 cr AP | 0:0:0:0 = 0 | Cr vb | 0:0:0:0 = 0 | Tub | 0:0:0:0 = 0 |  |  |
| T4-T5-Rib 5 | 9 | 0:0:0:0 = 0 | Both | 2:0:0:0 = 2 | All | 0:0:0:0 = 0 | Both | 2:0:0:0 = 2 | All | 2:0:0:0 = 2 | Both | 2:0:0:0 = 2 | 17 | 26 |
|  |  |  |  | 0:1:0:0 = 1 | Cd vb | 1:0:0:0 = 1 | T5 cd AP | 2:1:0:0 = 3 | Cd vb | 1:0:0:0 = 1 |  |  |  |  |
|  |  | 0:0:0:0 = 0 | Vb | 1:0:0:0 = 1 | Rib 6 |  |  |  | Rib 6 | 3:0:0:0 = 3 | Vb | 0:0:0:0 = 0 |  |  |
|  |  | 0:0:0:0 = 0 | Tub | 0:1:0:0 = 1 | Cr vb | 0:0:0:0 = 0 | T6 cr AP | 0:0:0:0 = 0 | Cr vb | 0:0:0:0 = 0 | Tub | 1:0:0:0 = 1 |  |  |
| T5-T6-Rib 6 | 6 | 0:0:0:0 = 0 | Both | 2:0:0:0 = 2 | All | 0:0:0:0 = 0 | Both | 0:0:0:0 = 0 | All | 2:0:0:0 = 2 | Both | 0:0:0:0 = 0 | 10 | 16 |
|  |  |  |  | 0:0:0:0 = 0 | Cd vb | 1:0:0:0 = 1 | T6 cd AP | 0:1:0:0 = 1 | Cd vb | 3:0:0:0 = 3 |  |  |  |  |
|  |  | 0:0:0:0 = 0 | Vb | 1:0:0:0 = 1 | Rib 7 |  |  |  | Rib 7 | 0:0:0:0 = 0 | Vb | 0:0:0:0 = 0 |  |  |
|  |  | 0:0:0:0 = 0 | Tub | 0:1:0:0 = 1 | Cr vb | 0:0:0:0 = 0 | T7 cr AP | 0:0:0:0 = 0 | Cr vb | 0:0:0:0 = 0 | Tub | 0:0:0:0 = 0 |  |  |
| T6-T7-Rib 7 | 3 | 0:0:0:0 = 0 | Both | 0:0:0:0 = 0 | All | 0:0:0:0 = 0 | Both | 0:0:0:0 = 0 | All | 2:0:0:0 = 2 | Both | 2:0:0:0 = 2 | 8 | 11 |
|  |  |  |  | 0:0:0:0 = 0 | Cd vb | 1:1:0:0 = 2 | T7 cd AP | 2:1:0:0 = 3 | Cd vb | 1:0:0:0 = 1 |  |  |  |  |
|  |  | 0:0:0:0 = 0 | Vb | 0:0:1:0 = 1 | Rib 8 |  |  |  | Rib 8 | 2:0:0:0 = 2 | Vb | 0:0:0:0 = 0 |  |  |
|  |  | 0:0:0:0 = 0 | Tub | 1:0:0:0 = 1 | Cr vb | 0:0:0:0 = 0 | T8 cr AP | 0:0:0:0 = 0 | Cr vb | 0:0:0:0 = 0 | Tub | 0:0:0:0 = 0 |  |  |
| T7-T8-Rib 8 | 6 | 0:0:0:0 = 0 | Both | 0:0:0:0 = 0 | All | 0:2:0:0 = 2 | Both | 0:0:0:0 = 0 | All | 0:0:0:2 = 2 | Both | 0:0:0:0 = 0 | 8 | 14 |

|  |  |  |  | 0:0:2:0 = 2 | Cd vb | 1:0:0:0 = 1 | T8 cd AP | 1:0:0:0 = 1 | Cd vb | 0:0:0:0 = 0 |  |  |  |  |
| --- | --- | --- | --- | --- | --- | --- | --- | --- | --- | --- | --- | --- | --- | --- |
|  |  | 0:0:0:0 = 0 | Vb | 0:0:0:0 = 0 | Rib 9 |  |  |  | Rib 9 | 2:0:0:0 = 2 | Vb | 0:0:0:0 = 0 |  |  |
|  |  | 1:0:0:0 = 1 | Tub | 0:0:0:0 = 0 | Cr vb | 1:0:0:0 = 1 | T9 cr AP | 0:0:0:0 = 0 | Cr vb | 0:0:0:0 = 0 | Tub | 0:0:0:0 = 0 |  |  |
| T8-T9-Rib 9 | 11 | 0:0:0:0 = 0 | Both | 2:0:0:0 = 2 | All | 0:2:2:0 = 4 | Both | 0:0:0:0 = 0 | All | 0:0:0:0 = 0 | Both | 0:0:0:0 = 0 | 3 | 14 |
|  |  |  |  | 2:1:0:0 = 3 | Cd vb | 1:1:0:0 = 2 | T9 cd AP | 1:0:0:1 = 2 | Cd vb | 1:0:0:0 = 1 |  |  |  |  |
|  |  | 1:0:0:0 = 1 | Vb | 0:0:0:0 = 0 | Rib 10 |  |  |  | Rib 10 | 0:0:0:0 = 0 | Vb | 0:0:0:0 = 0 |  |  |
|  |  | 0:0:0:0 = 0 | Tub | 0:0:0:0 = 0 | Cr vb | 1:0:0:0 = 1 | T10 cr AP | 1:1:0:0 = 2 | Cr vb | 0:0:0:0 = 0 | Tub | 0:0:0:0 = 0 |  |  |
| T9-T10-Rib 10 | 7 | 0:0:0:0 = 0 | Both | 0:0:0:0 = 0 | All | 0:0:0:0 = 0 | Both | 0:2:0:2 = 4 | All | 4:0:0:0 = 4 | Both | 2:0:0:0 = 2 | 15 | 22 |
|  |  |  |  | 0:0:0:0 = 0 | Cd vb | 4:1:0:0 = 5 | T10 cd AP | 5:0:0:0 = 5 | Cd vb | 0:0:0:0 = 0 |  |  |  |  |
|  |  | 0:0:0:0 = 0 | Vb | 0:0:0:0 = 0 | Rib 11 |  |  |  | Rib 11 | 0:0:0:0 = 0 | Vb | 0:0:0:0 = 0 |  |  |
|  |  | 0:0:0:0 = 0 | Tub | 1:0:0:0 = 1 | Cr vb | 0:0:0:0 = 0 | T11 cr AP | 0:0:0:0 = 0 | Cr vb | 0:0:0:0 = 0 | Tub | 0:0:0:0 = 0 |  |  |
| T10-T11-Rib 11 | 12 | 0:0:0:0 = 0 | Both | 2:0:0:0 = 2 | All | 0:4:0:0 = 4 | Both | 4:0:0:0 = 4 | All | 2:0:0:0 = 2 | Both | 2:0:0:0 = 2 | 13 | 25 |
|  |  |  |  | 0:0:0:0 = 0 | Cd vb | 1:2:0:0 = 3 | T11 cd AP | 1:2:1:0 = 4 | Cd vb | 0:0:0:0 = 0 |  |  |  |  |
|  |  | 0:0:0:0 = 0 | Vb | 1:0:0:0 = 1 | Rib 12 |  |  |  | Rib 12 | 2:0:0:0 = 2 | Vb | 0:0:0:0 = 0 |  |  |
|  |  | 0:0:0:0 = 0 | Tub | 0:0:0:0 = 0 | Cr vb | 0:0:0:0 = 0 | T12 cr AP | 0:0:0:0 = 0 | Cr vb | 0:0:0:0 = 0 | Tub | 0:0:0:0 = 0 |  |  |
| T11-T12-Rib 12 | 8 | 0:0:0:0 = 0 | Both | 0:0:0:0 = 0 | All | 1:3:0:0 = 4 | Both | 2:0:0:0 = 2 | All | 0:0:0:0 = 0 | Both | 0:0:0:0 = 0 | 8 | 16 |
|  |  |  |  | 0:0:0:0 = 0 | Cd vb | 0:1:0:0 = 1 | T12 cd AP | 2:1:2:0 = 5 | Cd vb | 0:0:0:0 = 0 |  |  |  |  |
|  |  | 1:0:0:0 = 1 | Vb | 1:0:0:0 = 1 | Rib 13 |  |  |  | Rib 13 | 1:0:0:0 = 1 | Vb | 0:0:0:0 = 0 |  |  |
|  |  | 0:0:0:0 = 0 | Tub | 0:1:0:0 = 1 | Cr vb | 0:0:0:0 = 0 | T13 cr AP | 0:0:0:0 = 0 | Cr vb | 0:0:0:0 = 0 | Tub | 0:0:0:0 = 0 |  |  |
| T12-T13-Rib 13 | 10 | 0:2:0:0 0 2 | Both | 2:0:0:0 = 2 | All | 2:0:0:0 = 2 | Both | 0:0:0:0 = 0 | All | 0:0:0:0 = 0 | Both | 2:0:0:0 = 2 | 8 | 18 |
|  |  |  |  | 0:0:0:0 = 0 | Cd vb | 2:0:1:0 = 3 | T13 cd AP | 5:3:0:0 = 8 | Cd vb | 0:0:0:0 = 0 |  |  |  |  |
|  |  | 0:0:0:0 = 0 | Vb | 0:0:0:0 = 0 | Rib 14 |  |  |  | Rib 14 | 0:0:0:0 = 0 | Vb | 0:0:0:0 = 0 |  |  |
|  |  | 0:0:0:0 = 0 | Tub | 0:0:0:0 = 0 | Cr vb | 1:0:0:0 = 1 | T14 cr AP | 1:0:0:0 = 1 | Cr vb | 0:0:0:0 = 0 | Tub | 0:0:0:0 = 0 |  |  |
| T13-T14-Rib 14 | 8 | 0:0:0:0 = 0 | Both | 2:0:0:0 = 2 | All | 0:2:0:0 = 2 | Both | 4:2:0:0 = 6 | All | 2:0:0:0 = 2 | Both | 0:0:0:0 = 0 | 17 | 25 |
|  |  |  |  | 0:0:0:0 = 0 | Cd vb | 6:3:0:0 = 9 | T14 cd AP | 8:4:0:0 = 12 | Cd vb | 0:0:0:0 = 0 |  |  |  |  |
|  |  | 0:0:0:0 = 0 | Vb | 0:0:0:0 = 0 | Rib 15 |  |  |  | Rib 15 | 0:0:0:0 = 0 | Vb | 0:0:0:0 = 0 |  |  |
|  |  | 0:0:0:0 = 0 | Tub | 0:0:0:0 = 0 | Cr vb | 1:0:0:0 = 1 | T15 cr AP | 0:0:0:0 = 0 | Cr vb | 0:0:0:0 = 0 | Tub | 0:0:0:0 = 0 |  |  |
| T14-T15-Rib 15 | 12 | 0:0:0:0 = 0 | Both | 0:0:0:0 = 0 | All | 0:2:0:0 = 2 | Both | 1:3:0:0 = 4 | All | 0:0:0:0 = 0 | Both | 0:0:0:0 = 0 | 16 | 28 |
|  |  |  |  | 0:0:0:0 = 0 | Cd vb | 4:1:2:0 = 7 | T15 cd AP | 3:1:0:0 = 4 | Cd vb | 0:0:0:0 = 0 |  |  |  |  |
|  |  | 0:0:0:0 = 0 | Vb | 0:0:0:0 = 0 | Rib 16 |  |  |  | Rib 16 | 0:0:0:0 = 0 | Vb | 0:0:0:0 = 0 |  |  |
|  |  | 0:0:0:0 = 0 | Tub | 0:0:0:0 = 0 | Cr vb | 0:0:0:0 = 0 | T16 cr AP | 1:0:0:0 = 1 | Cr vb | 0:0:0:0 = 0 | Tub | 0:0:0:0 = 0 |  |  |
| T15-T16-Rib 16 | 7 | 0:0:0:0 = 0 | Both | 0:0:0:0 = 0 | All | 0:0:0:0 = 0 | Both | 0:0:0:0 = 0 | All | 0:0:0:0 = 0 | Both | 0:0:0:0 = 0 | 5 | 12 |
| Sum thoracic segment | 161 | 6:6:0:0 |  | 39:16:8:0 |  | 46:29:9:2 | 236:91:27:7  148 cr: 30 rib: 31 cd: 152 both/all | 67:29:4:3 |  | 61:11:6:2 |  | 17:0:0:0 | 200 | 361 |

^1^Osteochondrosis: osteochondrosis dissecans: cyst: other = sum. ^2^Cd: Caudal. ^3^vb/Vb: Vertebra. ^4^AP: Articular process. ^5^Tub: Tubercle.

**c.** Lumbar segment (Four pigs had five lumbar vertebrae, counted from caudal to cranial)

| **Articulation level** | **Sum left side** | **Left side^1^** | **Articular process** | **Right side^1^** | **Sum right side** | **Sum left and right** |
| --- | --- | --- | --- | --- | --- | --- |
|  |  | 6:2:1:0 = 9 | T_ult_^2^ caudal AP^3^ | 9:2:0:0 = 11 |  |  |
|  |  | 1:1:0:0 = 2 | L1 cranial AP | 1:0:1:0 = 2 |  |  |
| T-L^4^ joint | 29 | 11:7:0:0 = 18 | Both | 10:4:0:0 = 14 | 27 | 56 |
|  |  | 7:6:0:1 =14 | L1 caudal AP | 11:2:3:0 =16 |  |  |
|  |  | 1:0:0:0 = 1 | L2 cranial AP | 0:1:1:0 = 2 |  |  |
| L1-L2 | 29 | 9:5:0:0 = 14 | Both | 3:7:0:0 = 10 | 28 | 57 |
|  |  | 11:1:0:0 = 12 | L2 caudal AP | 12:2:0:0 = 14 |  |  |
|  |  | 1:0:0:0 = 1 | L3 cranial AP | 1:1:0:0 = 2 |  |  |
| L2-L3 | 37 | 14:8:2:0 = 24 | Both | 5:13:2:0 =20 | 36 | 73 |
|  |  | 9:2:1:0 =12 | L3 caudal AP | 11:3:0:0 = 14 |  |  |
|  |  | 1:0:1:0 = 2 | L4 cranial AP | 1:0:0:0 = 1 |  |  |
| L3-L4 | 32 | 8:8:0:2 = 18 | Both | 18:2:2:0 = 22 | 37 | 69 |
|  |  | 11:1:0:0 = 12 | L4 caudal AP | 8:1:4:0 = 13 |  |  |
|  |  | 4:0:1:0 = 5 | L5 cranial AP | 2:1:0:0 = 3 |  |  |
| L4-L5 | 29 | 8:4:0:0 = 12 | Both | 8:8:4:0 = 20 | 36 | 65 |
|  |  | 6:6:0:0 = 12 | L5 caudal AP | 7:1:1:0 = 9 |  |  |
|  |  | 1:0:0:0 = 1 | L6 cranial AP | 2:0:0:0 = 2 |  |  |
| L5-L6 | 27 | 10:2:2:0 = 14 | Both | 17:1:0:0 = 18 | 29 | 56 |
|  |  | 5:1:0:0 = 6 | L6 caudal AP | 1:2:1:0 = 4 |  |  |
|  |  | 3:0:0:0 = 3 | S1 cranial AP | 3:0:0:0 = 3 |  |  |
| L6-S1 | 11 | 2:0:0:0 = 2 | Both | 4:2:0:0 = 6 | 13 | 24 |
| Sum lumbar segment | 194 | 129:54:9:2 | 263:107:28:2  158 cranial,  30 caudal,  212 both | 134:53:19:0 | 206 | 400 |

^1^Osteochondrosis: osteochondrosis dissecans: cyst: other = sum.^2^T_ult_: Ultimate thoracic vertebra. ^3^AP: Articular process. ^4^T-L: Thoraco-lumbar.
