# Supplementary material for: Osteochondrosis and other lesions in all intervertebral, articular process and rib joints from occiput to sacrum in pigs with poor back conformation, and relationship to juvenile kyphosis
Source: BMC Vet Res. 2022 Jan 18;18:44. doi: 10.1186/s12917-021-03091-6 (PMC8764802; doi:10.1186/s12917-021-03091-6)
Supplement: Supplementary file 3 — Additional file 3: Supplemental Table 3. Elbow and stifle osteochondrosis in the study population. [file 12917_2021_3091_MOESM3_ESM.docx]

**Supplemental Table 3.** Elbow and stifle osteochondrosis in study population

| **Pig** | **Elbow** | | | | **Stifle** | | | |  |
| --- | --- | --- | --- | --- | --- | --- | --- | --- | --- |
|  | **Right** | | **Left** | | **Right** | | **Left** | |  |
|  | **Lateral** | **Medial** | **Medial** | **Lateral** | **Lateral** | **Medial** | **Medial** | **Lateral** | **SUM** |
| 1 | - | + | - | + | + | + | + | - | 5 |
| 2* | - | - | - | + | - | + | + | + | 4 |
| 3 | - | - | - | + | + | + | + | - | 4 |
| 4 | - | - | - | - | + | + | + | + | 4 |
| 5 | - | + | - | - | + | + | + | + | 5 |
| 6* | + | - | - | - | + | + | + | + | 5 |
| 7* | - | - | - | - | - | + | - | + | 2 |
| 8 | + | + | - | + | - | - | - | - | 3 |
| 9 | - | + | - | - | - | + | + | + | 4 |
| 10* | - | - | - | - | + | + | + | + | 4 |
| 11 | + | + | - | - | - | + | + | - | 4 |
| 12* | - | - | - | + | - | + | + | + | 4 |
| 13 | + | + | + | - | + | + | + | - | 6 |
| 14 | - | - | - | - | - | - | + | + | 2 |
| 15* | - | - | - | + | - | - | - | - | 1 |
| 16* | - | - | - | - | - | + | + | - | 2 |
| 17 | - | - | - | - | + | + | + | - | 3 |
| 18* | + | + | - | + | - | - | + | + | 5 |
| 19 | + | + | - | + | - | + | + | + | 6 |
| 20 | - | + | + | - | + | + | + | + | 6 |
| 21 | + | + | + OA^1^ | + OA | + | + | + | + | 8 |
| 22* | - | + | - | + | - | + | + | + | 5 |
| 23* | + | - | - | + | - | - | - | - | 2 |
| 24 | + | + | + | - | - | + | + | + | 6 |
| 25* | + | - | - | - | + | + | + | + | 5 |
| 26* | - | + | + | - | - | + | + | + | 5 |
| 27* | - | - | + | - | + | + | + | + | 5 |
| 28* | + | - | - | - | - | - | - | - | 1 |
| 29* | - | + | + | - | + | + | + | - | 5 |
| 30* | - | + | - | - | - | + | + | - | 3 |
| 31 | - | - | - | - | + | + | - | - | 2 |
| 32 | - | - | + | - | - | + | + | - | 3 |
| 33* | - | + | + | - | - | + | - | - | 3 |
| 34 | - | + | - | - | + | + | + | - | 4 |
| 35 | - | - | + | + | + | + | + | + | 6 |
| 36 | - | + | - | - | + | + | + | + | 5 |
| 37* | + | + | - | + | + | + | + | + | 7 |
| **Lateral-**  **Medial** | **12** | **19** | **10** | **13** | **18** | **31** | **30** | **21** | **154** |
| **Right-**  **left** | **31** | | **23** | | **49** | | **51** | | **Mean: 4.16** |
| **Sum** | **54** | | | | **100** | | | | **Median: 4** |
|  |  | | | |  | | | | **Range: 1-8** |

*Pigs that received medical treatments are labelled with an asterisk. ^1^OA: Osteoarthritis.
