# Supplementary material for: Osteochondrosis and other lesions in all intervertebral, articular process and rib joints from occiput to sacrum in pigs with poor back conformation, and relationship to juvenile kyphosis
Source: BMC Vet Res. 2022 Jan 18;18:44. doi: 10.1186/s12917-021-03091-6 (PMC8764802; doi:10.1186/s12917-021-03091-6)
Supplement: Supplementary file 4 — Additional file 4: Supplemental Figure 1. Ventral vertebral lesion represents ischaemic chondronecrosis in a 12 kg piglet. a. The secondary ossification centres (2°) are absent caudo-ventrally in T8 and cranio-ventrally in T9. There is a lesion centred at the junction between T8-T9; higher magnification of the tissue inside the dashed box is shown in b. b. The lesion consists of necrotic cartilage canal vessels (asterisks), surrounded by necrotic chondrocytes (within dashed lines), representing ischaemic chondronecrosis identical to limb osteochondrosis. Viable chondrocytes (arrows) on the margin of the area of chondronecrosis are proliferating. a. The intervertebral disc appears to be absent, prompting the question of whether the lesion represents failure of the blood supply to growth cartilage, to the intervertebral disc, or both. a-b. Para-sagittal histological section from T8-T9 of a 12 kg mixed-breed piglet; a. 10x; b. 100x magnification, haematoxylin and eosin. [file 12917_2021_3091_MOESM4_ESM.docx]

**Supplemental figure 1.** Ventral vertebral lesion represents ischaemic chondronecrosis in a 12 kg piglet.


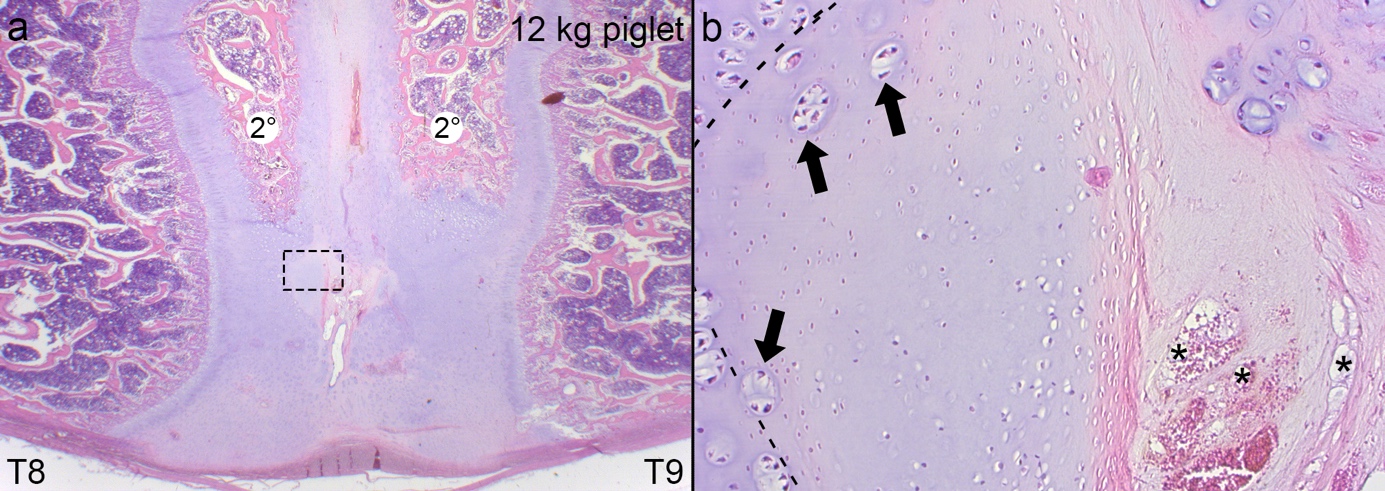


**a.** The secondary ossification centres (2º) are absent caudo-ventrally in T8 and cranio-ventrally in T9. There is a lesion centred at the junction between T8-T9; higher magnification of the tissue inside the dashed box is shown in **b.** **b.** The lesion consists of necrotic cartilage canal vessels (asterisks), surrounded by necrotic chondrocytes (within dashed lines), representing ischaemic chondronecrosis identical to limb osteochondrosis. Viable chondrocytes (arrows) on the margin of the area of chondronecrosis are proliferating . **a.** The intervertebral disc appears to be absent, prompting the question of whether the lesion represents failure of the blood supply to growth cartilage, to the intervertebral disc, or both.

**a-b.** Para-sagittal histological section from T8-T9 of a 12 kg mixed-breed piglet; **e.** 10x; **f.** 100x magnification, haematoxylin and eosin.
